# The Transcription Factors Snail and Slug Activate the Transforming Growth Factor-Beta Signaling Pathway in Breast Cancer

**DOI:** 10.1371/journal.pone.0026514

**Published:** 2011-10-20

**Authors:** Archana Dhasarathy, Dhiral Phadke, Deepak Mav, Ruchir R. Shah, Paul A. Wade

**Affiliations:** 1 Laboratory of Molecular Carcinogenesis, National Institute of Environmental Health Sciences (NIEHS), Research Triangle Park, North Carolina, United States of America; 2 SRA International Inc., Research Triangle Park, North Carolina, United States of America; National Univesity of Singapore, Singapore

## Abstract

The transcriptional repressors Snail and Slug are situated at the core of several signaling pathways proposed to mediate epithelial to mesenchymal transition or EMT, which has been implicated in tumor metastasis. EMT involves an alteration from an organized, epithelial cell structure to a mesenchymal, invasive and migratory phenotype. In order to obtain a global view of the impact of Snail and Slug expression, we performed a microarray experiment using the MCF-7 breast cancer cell line, which does not express detectable levels of Snail or Slug. MCF-7 cells were infected with Snail, Slug or control adenovirus, and RNA samples isolated at various time points were analyzed across all transcripts. Our analyses indicated that Snail and Slug regulate many genes in common, but also have distinct sets of gene targets. Gene set enrichment analyses indicated that Snail and Slug directed the transcriptome of MCF-7 cells from a luminal towards a more complex pattern that includes many features of the claudin-low breast cancer signature. Of particular interest, genes involved in the TGF-beta signaling pathway are upregulated, while genes responsible for a differentiated morphology are downregulated following Snail or Slug expression. Further we noticed increased histone acetylation at the promoter region of the transforming growth factor beta-receptor II (*TGFBR2*) gene following Snail or Slug expression. Inhibition of the TGF-beta signaling pathway using selective small-molecule inhibitors following Snail or Slug addition resulted in decreased cell migration with no impact on the repression of cell junction molecules by Snail and Slug. We propose that there are two regulatory modules embedded within EMT: one that involves repression of cell junction molecules, and the other involving cell migration via TGF-beta and/or other pathways.

## Introduction

Breast cancer is the most frequently diagnosed malignancy in women worldwide [reviewed in [1]]. In recent years, prognosis for breast cancer has improved as a result of advances in diagnosis and treatment. Nevertheless, tumor dormancy after treatment followed by local, regional or distant recurrence is a leading cause of breast cancer mortality [reviewed in [1]]. Clinically, advanced-stage breast cancer is characterized by metastasis, a multi-step process postulated to involve cancer cell invasion, proliferation, and eventual survival in distant tissues following transport by the circulatory system [1]. An early developmental phenomenon known as ‘Epithelial to Mesenchymal Transition’ or EMT, which results in the acquisition of an invasive, mesenchymal phenotype by epithelial cells, has been postulated to play an important role in cancer metastasis [reviewed in [2]]. Recently, cell-fate mapping strategies in mouse models of mammary tumors [3] provided direct evidence for EMT.

Several proteins that are involved in EMT during early embryonic development have come under close scrutiny in cancer cell programs. In particular, the role of the Snail family of zinc finger proteins in EMT and cancer has been highlighted in several publications [reviewed in [4]]. These proteins effect changes in gene expression that are required for such important developmental processes like mesoderm formation, left-right identity and cell fate decisions [reviewed in [4]]. Responding to environmental cues, the highly related transcriptional repressors Snail (*SNAI1*) and Slug (*SNAI2*) are thought to act as master regulators, altering expression of a number of genes including E-cadherin (*CDH1*) [4], [5], [6], and contribute to physiological changes resulting in EMT [4], [7].

Despite the many similarities between Snail and Slug, there are clear differences in their biological functions. Combinatorial depletion of both factors by RNAi in an in vitro model of EMT led to a more dramatic phenotypic alteration than modulation of either factor alone [8]. Slug knockout mice can survive to adulthood, while Snail knockout mice die at gastrulation [reviewed in [4]]. Further, oncogene induction in an inducible mouse model of mammary adenocarcinoma led to robust activation of Snail, but not Slug, in the recurrent tumors that arose after withdrawal of induction and primary tumor regression [9]. Thus, while Snail and Slug collaborate towards a similar goal of enhancing tumor growth potential and induction of distant metastases, they might play important but distinct roles at each stage of the tumor dissemination process (i.e. migration, intravasation, transport to a distant site, extravasation, or adaptation and growth of the relocated cancer cells). These observations predict distinct roles for Snail and Slug, and are compatible with current models [10], [11], [12].

The TGF-beta family of signaling molecules has been implicated in the upregulation of Snail [13], [14] and Slug [15], and also in EMT [16], [17]. Additionally, TGF-beta dependent activation of Snail and Slug is thought to be mutually exclusive and context dependent [18]. The role of TGF-beta in cancer is highly complex: this pathway functions both in tumor suppression and in tumor promotion, and *TGFBR2* is frequently inactivated in breast cancer [ (reviewed in [19]], as it inhibits proliferation in normal breast cells [reviewed in [19]. Absence of *TGFBR2* and its downstream targets, including the Smad transcription factors, has been reported in different types of cancers [19], [20]. However, increased TGF-beta expression is positively correlated with breast and other cancers, as well as invasive lymph node metastases in breast cancer [10]. Smad3 and 4, downstream targets of *TGFBR2*, collaborate with Snail in downregulation of E-cadherin [21]. The TGF-beta pathway also induces Snail and Slug expression via the Smad and *HMGA2* proteins [22], [23].

In this study, we examined the transcriptional consequences of exogenous expression of Snail or Slug at the global level in a luminal breast cancer cell line, MCF7. We observed downregulation of transcripts integral to the luminal pattern of gene expression observed in primary breast tumors [24], [25]. In addition, we found upregulation of genes characteristic of the basal and claudin-low patterns [24], [25]. Further, we demonstrated the upregulation of TGF-beta signaling pathway members following Snail and Slug expression. Pharmacologic blockade of TGF-beta signaling decreased the migratory properties of cells following Snail and Slug expression. Nevertheless, inhibition of TGF-beta does not affect repression of cell junctional molecules by Snail and Slug. We therefore propose that the EMT phenotype is a sum of at least two distinct alterations: (a) changes in the transcriptional program from a luminal to a more basal or claudin-low subtype induced by transcription factors like Snail and Slug, and (b) a morphologic change resulting in increased migration induced by TGF-beta and other pathways.

## Results

### Snail and Slug regulate the expression of several genes in common, but exhibit specificity in some of their gene targets

**Figure 1:**
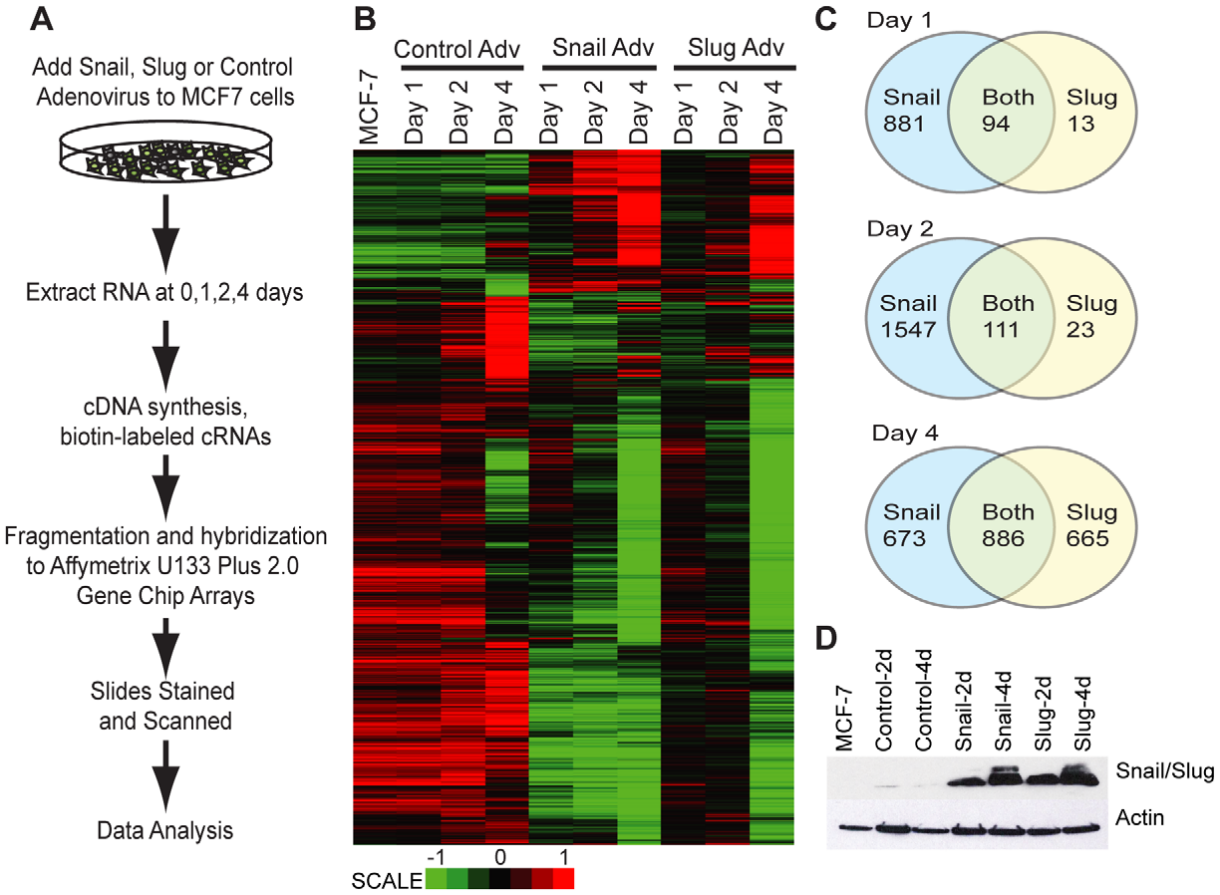
Snail and Slug regulate common and unique sets of genes in breast cancer cells. A) Schematic representation of microarray experiment. B) Heat map depicting probes with absolute fold >2 and adjusted p-value<0.01 on Day4 after Snail and Slug expression, shown over time. C) Venn diagrams showing the total number of genes that change following Snail or Slug expression, with absolute fold >2 and adjusted p-value<0.01. D) Immunoblots showing the expression of Snail, Slug or Actin in MCF-7 (untreated) and following 2–4 days of adenovirus expression.

To obtain a global view of the genome-wide impact of Snail or Slug expression in a luminal breast cancer cell model (MCF-7), we isolated RNA from MCF-7 cells 0,1, 2 and 4 days following infection with control, Snail or Slug adenovirus (figures 1A and D) and examined transcriptome changes using Affymetrix arrays. After normalization, we filtered the data for genes with a two-fold or higher change in expression (up or down) and an adjusted p-value (false discovery rate) of 0.01 or lower. Using this filtered set of genes, we observed alterations in steady state levels of many transcripts following Snail or Slug expression relative to the day 0 and control adenovirus expressing cells (figure 1B and C). PCA analysis (Figure S1) indicated that the replicates clustered together with no outliers, and there were clear differences between the control and Snail/Slug treatments for each day.

Relative to the control-infected cells, we found significant changes in the levels of 975 genes following Snail expression, but only 107 for Slug (Figure 1C and Table S1) after one day of infection with the adenovirus. On day 2, Snail expression resulted in downregulation of 1281 and upregulation of 377 genes, while Slug expression on day 2 caused downregulation of 69 and upregulation of 65 genes (Figure 1C and Table S1). By day 4, Snail-treated cells had changes in the expression of 1559 genes in total, and 1551 genes for Slug (Figure 1C and Table S1). In both cases, there are more downregulated genes than upregulated, which agrees with published literature arguing a repressive role for these proteins [4]. At the earlier time points, Snail-treated samples had more gene expression changes relative to Slug, but both achieved similar gene expression profiles by day 4 (Figure 1B). Immunoblotting analysis (Figure 1D) revealed that there were similar levels of Snail and Slug protein expression; therefore, the difference in the number of genes regulated by Snail and Slug at earlier time points might reflect differences in binding affinity of Snail and Slug, and/or the availability of cofactors. Snail and Slug did not influence the expression of each other; therefore the changes seen here are solely due to either Snail or Slug (Figure S2).

Many of the genes that changed in expression were common to both Snail and Slug; however, as reported in other systems [26], [27], there were also unique categories regulated by Snail or Slug (Figure 1C and Table S1). We validated some of the targets: those that were previously described for their importance in cancer, like *CDH1* [6], [28], *OCLN* [29] and *ESR1* [30] (Figure 3B) and some of the genes from our microarray analysis that were differentially regulated by Snail and Slug (Figure S3). Among the differentially expressed genes were *ITGA2*, *EXPH5* and *SGK3* for Snail and *G6PC3*, *TCF3* and *SERPINE1* for Slug (Figure S3A and B). We categorized the sets of genes that changed following Snail and Slug expression for each time point using Ingenuity pathway analysis (IPA) (File S1). As expected, genes involved in cell-cell signaling and interaction, cell movement, development, growth and proliferation, and cell death were among the top pathways common to both Snail and Slug (File S1). The unique biological pathways we found included p53 signaling, neuregulin signaling, interferon signaling, and ERK5 signaling for Slug; and steroid and glycosphingolipid biosynthesis, tight junction signaling and aryl hydrocarbon receptor signaling for Snail (File S1). The transcriptional signatures induced by both Snail and Slug suggest that each of these transcription factors induces genes integral to migration, invasion and metastasis, while the unique subsets of genes that are changed may reflect subtle alterations in the biological program of cancer cells reflective of signaling environment or other complex biological parameters.

### Snail and Slug decrease expression of differentiation-specific genes that contribute to the breast epithelial morphology while increasing TGF-beta family expression

**Figure 2:**
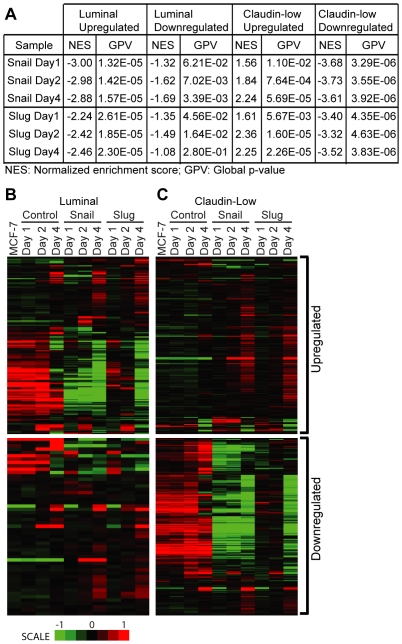
Snail and Slug cause changes in expression that most resemble the claudin-low class of breast tumors. GSEA analysis (A) comparing the genes in the microarray samples to those that were upregulated (B and C, top panels) or downregulated (B and C, bottom panels) from the claudin-low class (B) and luminal class (C) described in [24]. See text for details. NES = Normalized enrichment score, GPV = Global p-value.

Breast cancers have been classified into 5 molecular classes: normal breast like, claudin-low, basal-like, Her2-enriched and luminal [24], [25]. MCF-7 cells closely resemble the luminal gene expression pattern [25], [31], [32]. We asked whether the molecular signature of MCF-7 cells changes following Snail and/or Slug expression using GSEA, a computational method that assesses whether an a priori defined set of genes shows statistically significant, concordant differences between two biological states [33]. We included gene sets representing claudin-low, basal, Her-2, luminal and normal breast-like expression patterns within the standard GSEA framework (see methods for details) [24]. Our analyses revealed that the claudin-low signature was most enriched and displayed the most significant global p-value relative to the other categories of breast cancer cells (Figure 2 and Figure S4). These results demonstrate that the Snail and Slug expression in MCF-7 cells alters the expression pattern from luminal to a more complex pattern with features of the claudin-low subtype being statistically enriched (Figure 2). Further, a large number of genes with low-level expression in the claudin-low signature were downregulated in Snail and Slug expressing cells (Figure 2) consistent with their classification as transcriptional repressors.

**Figure 3:**
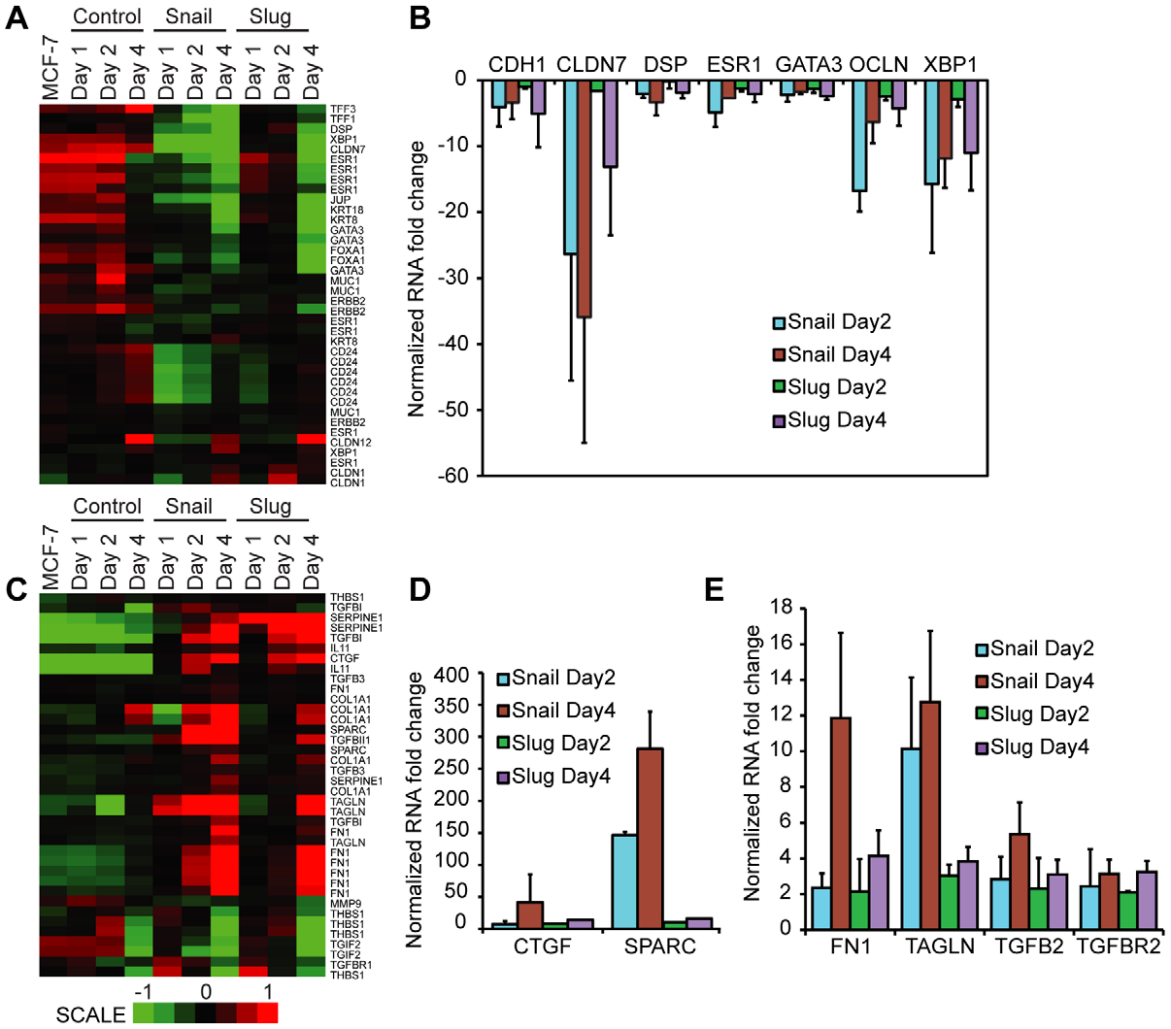
Snail and Slug expression in MCF-7 cells decreases the expression of differentiation markers, and increases the expression of TGF-beta pathway genes. Heat maps depicting (A) differentiated cell markers (C) TGF-b pathway probes following Snail and Slug expression. (B, D and E) RT-PCR of cDNA with real-time quantitation following normalization to 18 s, MCF-7 Day 0 and control adenovirus. The data represents the average of three independent biological replicates.

Similar to the claudin-low category of breast tumors, the CD44+ breast tumor cells also express low levels of differentiation specific markers [34]. In addition, the CD44+ cells have high levels of TGF-beta and stem cell genes [34]. To determine whether Snail and Slug expression elicit a similar pattern, we generated genesets representing the differentiated cell markers and TGF-beta cassette as described by Polyak and colleagues, and used it within the standard GSEA framework (see methods for details) [34]. Indeed, the upper tier of significant molecular gene signatures in our dataset using GSEA analysis included TGF-beta pathway genes (upregulated) and differentiation specific genes (downregulated) (Table 1 and Figure 3A and C).

**Table 1:** GSEA analysis.

	TGF-beta family markers			Differentiated Cell Markers		
Signatures	Normalized Enrichment Score (NES)	False Discovery Rate	Global p-value (GPV)	Normalized Enrichment Score (NES)	False Discovery Rate	Global p-value (GPV)
Snail Day 1	2.31	0.001	0.000006	−2.68	0.001	0.000023
Snail Day 2	2.59	0.001	0.000011	−2.59	0.001	0.000025
Snail Day 4	2.93	0.001	0.000005	−2.32	0.001	0.000063
Slug Day 1	2.69	0.001	0.000004	−2.34	0.001	0.000017
Slug Day 2	2.71	0.001	0.000004	−1.79	0.010	0.001320
Slug Day 4	2.48	0.001	0.000015	−2.35	0.001	0.000038

GSEA was performed using genesets consisting of differentiated cell markers and the TGF-beta genes described in [34]. The global p-value represents the tail probability from a density plot of normalized enrichment score values across all genesets and all permutations.

Next, we validated key genes revealed in the enrichment analysis by real-time RT-PCR. Both Snail and Slug expression in MCF-7 cells caused decreased expression of differentiation specific markers (Figure 3A and B) including *ESR1*, *OCLN* and *CDH1* [5], [28], [29], [30], [35]. Importantly, these genes contain putative Snail and/or Slug binding sites in their 5′ regions and have been previously shown to be direct targets of Snail and/or Slug [6], [30], [36]. In addition, TGF-beta pathway genes were upregulated following Snail and Slug expression (Figure 3C–E). Among these, *SPARC* (secreted protein, acidic, cysteine-rich) is a matrix-associated protein that influences changes in cell shape and synthesis of the extracellular matrix, and inhibits cell-cycle progression [37]. *SPARC* has been identified as a putative target of Snail and Slug [38], as well as TGF-beta [39]. Connective tissue growth factor (*CTGF*) has been reported to be selectively upregulated in fibroblasts following activation by TGF-beta [40], [41], [42]. It has been suggested that *CTGF* along with Interleukin 11 might influence cancer metastasis to the bone in breast cancer [43]. Transgelin, an actin-binding protein sensitive to changes in cell shape, is thought to be involved in cell migration [44], and is a direct downstream target of TGF-beta signaling [44]. Collectively our data indicate that expression of several genes in the TGF-beta family is upregulated following Snail and Slug expression.

### Increased histone acetylation at TGFBR2 locus following Snail and Slug expression

**Figure 4:**
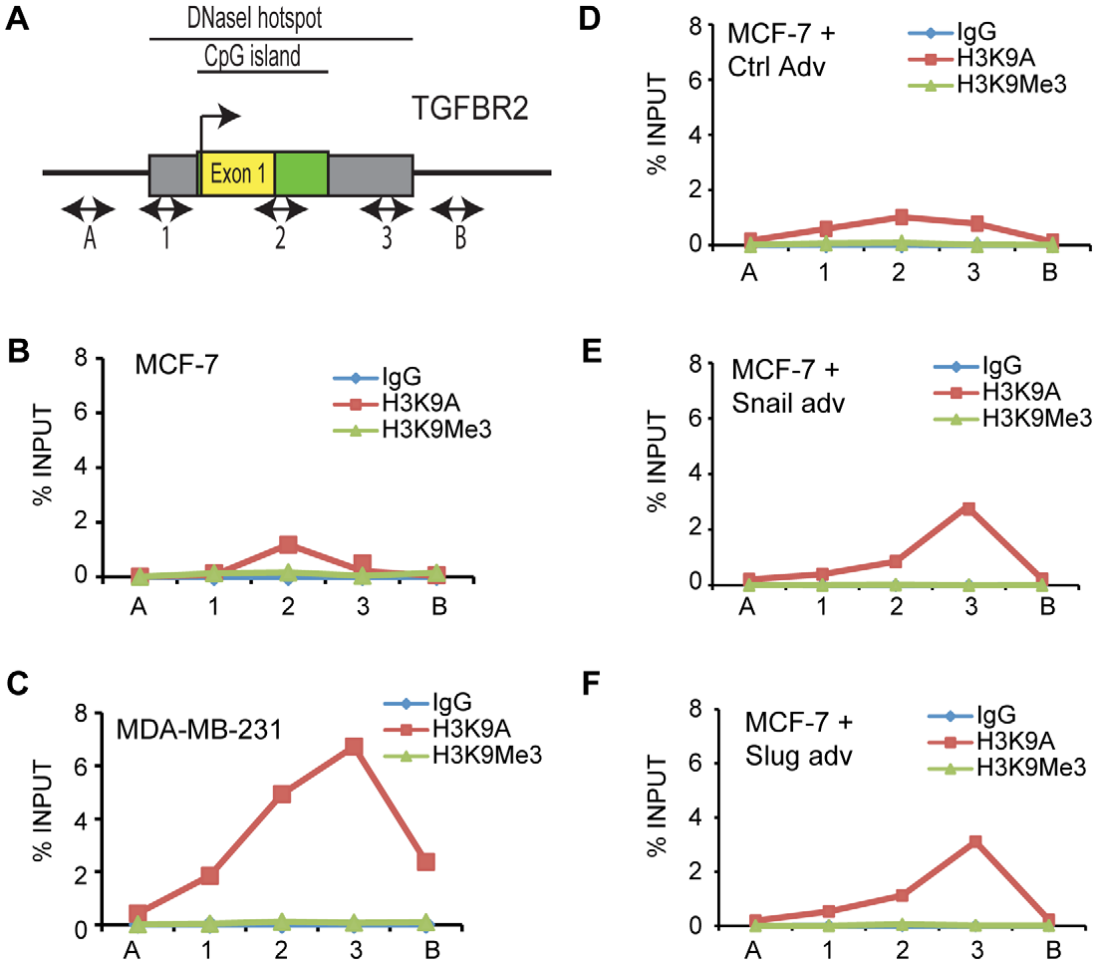
TGFBR2 shows distinct histone acetylation patterns in MCF7 and MDA231, and this changes upon addition of Snail and Slug. Primers for ChIP were designed across the TGFBR2 promoter region (A). Chromatin IP across the TGFBR2 promoter in MDA-MB-231 (C) demonstrated an increased amount of histone H3K9 acetylation compared to MCF-7 cells (B). Increased acetylation is also seen when Snail or Slug are expressed in MCF-7 cells (E and F), relative to the control-adenovirus infected cells (D). The data is representative of three independent biological replicates.

The presence of *TGFBR2* among genes upregulated following Snail and Slug expression was intriguing, as the TGF-beta signaling pathway is upstream of Snail and Slug and induces their expression [14], [45], [46]. MCF-7 cells do not express much *TGFBR2* mRNA relative to MDA-MB-231 (Figure S5). We utilized chromatin immunoprecipitation (ChIP) to examine the histone modification status across the *TGFBR2* promoter (Figure 4A) in MDA-MB-231 and MCF-7 cells (Figure 4B and C), using antibodies against acetylated Histone H3 Lysine 9 (H3K9Ac), which is associated with active chromatin, and trimethylated Histone H3 Lysine 9 (H3K9Me3), which is found in conjunction with silent chromatin [47]. While we observed neither significant acetylation nor trimethylation of histone H3K9 across the *TGFBR2* promoter in MCF-7 cells (Figure 4B), there was a substantial level of H3K9 acetylation across the promoter in MDA-MB-231 cells where the gene is active (Figure 4C). The highest degree of acetylation at *TGFBR2* in MDA-MB-231 cells is found in the vicinity of primer set 3, which maps near the end of the first exon (Figure 4A and C).

To determine whether the addition of Snail and Slug enables a more ‘open’ chromatin status at the *TGFBR2* promoter, we performed ChIP in MCF-7 cells following two days of Snail and Slug expression. Relative to control-treated cells, we saw an increase in H3K9 acetylation in Snail and Slug expressing MCF-7 cells (Figure 4 D–F) in a pattern similar to MDA-MB-231 cells, with the highest level around primer set 3. The level of acetylation following Snail or Slug expression is higher than in MCF-7 cells, but not as much as MDA-MB-231 cells (compare Figure 4C to 4E and F). To our knowledge, this is the first demonstration that addition of Snail and Slug can increase histone acetylation status of the*TGFBR2* promoter.

### Treatment with inhibitors of TGF-beta signaling reduces cell migration ability in response to Snail and Slug

Both TGF-beta signaling and Snail (or Slug) are known to induce EMT [14], [45], [46]. TGF-beta is also known to induce Snail and Slug expression, and our studies indicate that Snail and Slug can induce TGF-beta pathway genes. Therefore, we enquired whether we could mechanistically dissect the effects of Snail (or Slug) and TGF-beta in inducing EMT. To this end, we treated MCF-7 cells with control, Snail or Slug adenovirus, and either DMSO or one of two small molecule inhibitors: SB431542, a highly specific inhibitor of TGFBR2 [48], and LY364947, which inhibits both TGFBR1, and to a lesser degree, TGFBR2 [49]. After two days of treatment with the virus and inhibitors or DMSO as vehicle control, we determined whether inhibiting the TGF beta pathway influenced the cell migration ability induced by Snail and Slug. We used a Boyden chamber assay to evaluate cell migration through a membrane coated with human Collagen IV.

**Figure 5:**
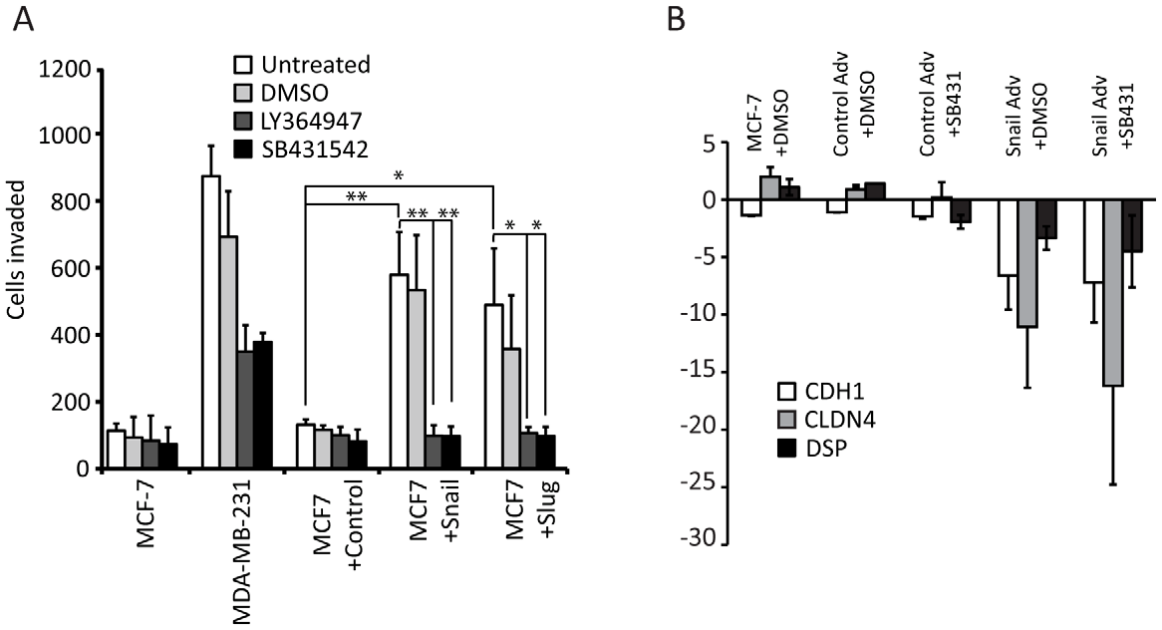
Treatment with a TGF-beta inhibitor reduces the migratory response to Snail and Slug. (A) Cell migration assay using MDA-MB-231, uninfected MCF-7 cells, and MCF-7 cells infected with control, Snail or Slug adenoviruses (white bars), treated with DMSO (light grey bars) or with 10 µM final of the TGF-beta inhibitors LY364947 (dark grey bars) or SB431542 (black bars) for 2 days. The X-axis represents the total number of cells in 10 fields. The data represents the average of three independent biological replicates (* T-test p-value ≤0.05, **T-test p-value ≤0.01). (B) Addition of TGF-beta inhibitor does not affect repression of cell junction molecules CDH1, DSP and CLDN4. MCF-7 cells were untreated or treated with control or Snail adenovirus, and with DMSO vehicle or SB431542 for 2 days. RNA was isolated and RT-PCR of cDNA with real-time quantitation was performed following normalization to GAPDH, and MCF-7 Day 0. The data represents the average of three independent biological replicates.

Compared to MCF-7, we found that MDA-MB-231 was highly migratory (Figure 5A). However, treatment with the inhibitors caused a decrease in the cell migration ability of MDA-MB-231 (Figure 5A). When we treated MCF-7 cells with the control adenovirus, it behaved similar to the MCF-7 cells without adenovirus treatment (Figure 5A). Addition of Snail or Slug increased the migration of MCF-7 by roughly three-fold relative to the control cells, while inhibition of TGF-beta signaling diminishes this response down to background levels (Figure 5A).

Components of the junctional complexes that join epithelial cells have been proposed to act as kinetic barriers to migratory and invasive growth in cancer [5], [50]. As the inhibitors employed here block acquisition of these properties, we asked whether they also impact repression of junctional complex components. Real-time PCR analysis of gene expression revealed that representative components of the adherens junction (E-cadherin), desmosome (Desmoplakin) or tight junctions (Claudin 4) were still repressed by Snail, despite the addition of TGF-beta inhibitors (Figure 5B). Thus, inhibition of the TGF-beta signaling pathway caused a decrease in cell migration that was not due to repression of cell junctional molecules by Snail.

## Discussion

EMT offers an attractive model to explain how epithelial cancer cells can change phenotype rapidly (yet reversibly) and migrate, leading to cancer metastasis [7]. The transcriptional repressors Snail and Slug are sufficient to induce EMT when expressed in epithelial systems [7], and so is triggering the TGF-beta signaling pathway [17], which can also stimulate Snail and Slug expression [14], [15], [46]. Here, we showed that Snail and Slug could in turn increase expression of the TGF-beta pathway members in a positive feedback mechanism, resulting in increased migratory properties of the cell. Further, we mechanistically dissected the roles for Snail and TGF-beta in eliciting EMT.

### Snail and Slug decrease the expression of differentiation-specific genes

Global gene expression profiling of normal and cancerous breast tissues have resulted in the molecular classification of breast cancer into luminal, basal, Her-2 enriched, claudin-low and normal breast-like groups [25]. These categories have been useful in predicting clinical parameters of disease including survival and response to treatment [25]. MCF-7 cells fall under the luminal A class, which is non-invasive, highly differentiated and positive for ER (Estrogen receptor) and PR (Progesterone receptor). Using GSEA, we found changes in a number of gene sets representing different breast cancer subtypes with significant p-values. However, when we performed GSEA by comparing the Control-treated cells to Snail and Slug treated cells, the Claudin-low gene set exhibited the most significant global p-value and the significantly large NES compared to all other cancer subtypes (Figures 2 and S4). These results indicate that the transformation in the transcriptome of MCF7 cells triggered by Snail/Slug expression, while complex, most closely resembles the claudin-low type (Figure 2). Snail and Slug expression in MCF-7 resulted in decreased expression of differentiation- specific molecules that make up the luminal A phenotype, and an increased claudin-low gene expression signature (Figures 2 and 3), which is triple negative (ER, PR and HER2 negative), highly invasive, and enriched with progenitor cell markers and a core EMT signature [51]. This is consistent with the model that indicates a close relationship between EMT and a CD44hi/CD24 lo/- stem cell phenotype (3, 50). Several genes in the basal class were also similarly up or downregulated following Snail and Slug expression, whereas the overlap was much less with genes in the HER2 class (Figure S4). Overall, this indicates that Snail and Slug drive the transcriptome profile of MCF-7 cells from a luminal towards a claudin-low/basal phenotype, which is associated with poorer prognosis in the clinic [24], [32].

### Snail and Slug expression upregulates TGF-beta pathway markers

Breast cells with a CD44^+^/CD24^−/lo^ phenotype show increased expression of the ‘TGF-beta cassette’ group of genes and progenitor markers [34]. Following Snail and Slug expression, we also observed a strong upregulation of the TGF-beta pathway markers (Figure 3C–E). Specifically, there was an increase in *CTGF*, *SPARC*, *TAGLN*, *TGFB2* and *TGFBR2* (Figure 3C–E), which are important in the TGF-beta signaling pathway [19], [20], [39], [40], [42], [44]. The transforming growth factor beta family of receptors is evolutionarily highly conserved, and is hypothesized to play a dual role in cancer progression [20], both as a tumor-suppressor in the normal mammary gland [19], and tumor promoter in mouse models [52] and others.

Our data imply that Snail and Slug upregulated *TGFBR2* expression (Figure 3), and this was accompanied by elevated levels of histone H3K9 acetylation (Figure 4), a mark that is associated with transcriptionally active chromatin [47] across the promoter region of *TGFBR2*. Whether Snail or Slug can directly or indirectly induce *TGFBR2* transcription remains to be studied.

### Snail and Slug induce an EMT phenotype at least in part through the action of TGF-beta signaling

The EMT-like phenotype induced by Snail and Slug appears to consist of two complementary processes: upregulation of TGF-beta and early progenitor cell markers, and a downregulation of the cell-cell adhesion and differentiation specific markers. The resulting so-called ‘metastable’ phenotype [7] results in a cell that is highly motile, resistant to apoptosis and has decreased cell-cell adhesion, all of which are important in achieving cell migration. These processes have been previously shown to be stimulated by both Snail and Slug, as well as by TGF-beta signaling. TGF-beta itself influences the actions of Snail by both increasing expression [14], [22] and promoting Snail binding at target promoters through the coactivator Smads [21]. Our data indicated that Snail and Slug can in turn upregulate the TGF-beta pathway genes (Figure 3C–E).

We attempted to mechanistically separate the actions of Snail/Slug and TGF-beta in inducing EMT by treating MCF-7 cells concurrently with Snail, Slug or control adenovirus and small molecule inhibitors that interfered with the TGF-beta signaling pathway (Figure 5A). Our experiments indicated that while Snail or Slug by themselves caused a roughly three-fold increase in cell migration relative to the control cells, inhibition of TGF-beta signaling diminished this response to near background levels (Figure 5A). However, cell junctional components are still repressed by Snail expression, despite inhibition of the TGF-beta pathway (Figure 5 B). This is reminiscent of other studies [53], [54] that proposed two components of EMT: a morphologic branch and a gene expression branch. Here, inhibition of TGF-beta does not appear to affect the gene repression induced by Snail (Figure 5 B); however, the ability of cells to migrate is affected (Figure 5 A). This could potentially occur through the TGF-beta- Par6 polarity pathway [53], [54], but further studies are needed to test this. We suggest that there are two branches of the EMT phenotype: one that involves the transcription program, i.e. repression of cell junction molecules by Snail or Slug, and the other involving cell migration via TGF-beta and/or other pathways.

### Snail, Slug and EMT in breast tumors

**Figure 6:**
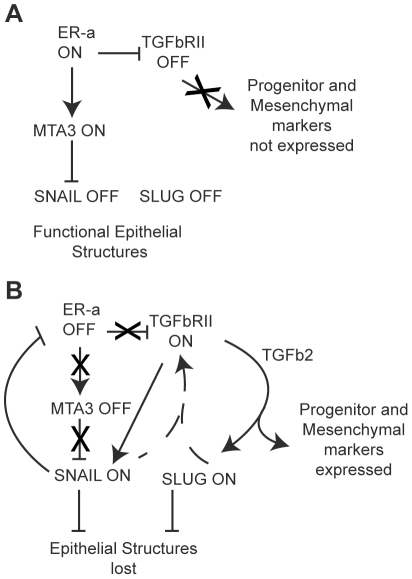
A role for Snail and Slug in upregulating TGF-beta during EMT. In normal breast cells, the TGF-beta pathway is active in keeping Estrogen receptor-alpha cells from proliferating. A) In cancer cells with a luminal phenotype (e.g. MCF-7), the TGF-beta pathway is downregulated, ER-alpha and MTA3 are expressed, and Snail expression is inhibited. This results in a differentiated and epithelial phenotype with well-preserved cell junctions, and the cells are adherent and non-migratory. B) During EMT, expression of Snail (and/or Slug) causes downregulation of ER-alpha and upregulation of the TGF-beta pathway. However, TGF-beta now serves a pro-metastatic role, and early progenitor markers are induced, resulting in a more ‘claudin-low’ morphology that is more mesenchymal and highly migratory.

EMT remains a highly controversial subject, with many pathologists rejecting the idea of a ‘transition’ in human breast tumors, while proponents of EMT have devised clever ways to demonstrate the phenomenon in mouse models [reviewed in [7]]. Whatever the case, there is no doubt that Snail and Slug remain positively correlated with highly migratory cells, both in the clinic and the laboratory [7], [10]. Thus, we can conceive of a model wherein epigenetic and/or environmental factors can trigger the expression of Snail and/or Slug in rapidly proliferating, differentiated tumor cells, resulting in (a) repression of differentiation specific genes and (b) activation of TGF-beta pathway and progenitor cell markers, leading to increased migration (Figure 6). The end result is a claudin-low phenotype, which is associated with highly invasive cancers that have a poor prognosis [24].

Anti-estrogenic compounds like tamoxifen, which target estrogen receptor signaling, are widely used for treating breast cancer in the clinic [2]. While this has proved effective in reducing tumor cell proliferation, loss of estrogen receptor signaling can inadvertently increase Snail and TGF-beta signaling [30], resulting in increased cell migration and metastasis. Indeed, some breast tumors develop resistance to anti-estrogenic treatment. Our results suggest that the cell migration induced by Snail or Slug expression could be checked by addition of TGF-beta signaling inhibitors (Figure 5A). Therefore, the sequential treatments with estrogenic antagonists to control tumor growth and TGF-beta inhibitors to prevent metastasis may have therapeutic benefit.

## Materials and Methods

### Cell culture and adenovirus methods

Human breast carcinoma cell lines MCF-7 and MDA-MB-231 were obtained from the American Type Culture Collection (Manassas, VA) and cultured in DMEM/F-12 medium supplemented with 10% FBS at 37 degrees C in 5% CO_2_. Adenoviruses were prepared as described previously [35].

### RNA isolation, microarray methodology and data analysis

Following 0,1,2 and 4 days of infection with Snail, Slug or control (GFP-only) adenovirus, RNA was isolated from MCF-7 cells using Trizol (Invitrogen), cleaned through Qiagen columns and resuspended in DEPC-treated water. The RNA was checked for quality and processed using standard hybridization protocols for Affymetrix Human Genome U133 Plus 2.0 GeneChip® arrays (Affymetrix, Santa Clara, CA). Arrays were scanned with the Affymetrix Scanner 3000 and data obtained using GeneChip® Operating Software (GCOS; Version 1.4.0.036). The .*cel* files were background corrected and normalized using Robust Multi-array Average (RMA) method [55]. Principal Component Analysis (PCA) was performed on the background corrected and normalized data, for all probes and samples using the programming language R (www.r-package.org) to characterize the variability present in the data. In order to identify differentially expressed probes, one-way analysis of variance (ANOVA) was used to determine if there was a statistical difference between the means of the untreated, control adenovirus-treated, and Snail or Slug adenovirus-treated groups at each time point. The resulting p-values were adjusted for multiple testing using the Benjamini Hochberg method (false discovery rate) [56]. Probes were ranked based on their adjusted p-values and fold change. Probes that displayed a fold change of two-fold or greater in either direction, along with adjusted p-values less than 0.01 were selected for further analysis. An unsupervised hierarchical clustering of samples based on the differentially expressed probes at day 4 using the normalized data and average linkage clustering resulted in a clear separation between the Snail or Slug samples compared to controls. The microarray data is available at the GEO database (http://www.ncbi.nlm.nih.gov/geo/query/acc.cgi), accession code GSE29672. All our data is MIAME compliant.

### GSEA analysis

Gene set enrichment analysis (GSEA) is a computational method that determines whether an a priori defined set of genes (referred as ‘gene sets’) shows statistically significant, concordant differences between two biological states (e.g. phenotypes). To perform such functional analysis of microarray data, the GSEA utilizes thousands of annotated gene sets hosted within the Molecular Signatures Database (MSigDB). In this study, GSEA [33] was performed using the gene sets available in the MSigDB v3.0, with an additional 12 gene sets added to the MSigDB. Specifically, we added 10 genesets corresponding to 5 molecular breast cancer signatures (up/down regulated in Basal, Claudin-low, Normal-like, Her2 and Luminal) [24], where the signatures comprised of genes with 2.5 or greater fold change in a particular breast cell type compared to all the rest [24]. In addition, we generated 2 genesets using the genes that comprise the differentiated cell markers and TGF-beta cassette described in [34]. We performed GSEA by comparing gene expression profile of Snail/Slug to Control at each day (day 1, 2 and 4). Gene sets were ranked by their global p-value. The global p-value represents the tail probability from a density plot of normalized enrichment score values across all gene sets and all permutations. The geneset enrichment statistics was derived using the probe-level signal-to-noise ratio measurements. The significance test of geneset enrichment statistic was performed using 1000 random probe/sample permutations. The normalized enrichment scores across all genesets and all permutations were used to compute false discovery rate and global p-value.. We generated heat maps to visualize the expression differences in Snail, Slug and controls for the set of genes mentioned above.

### Real-Time PCR validation

We performed RT-PCR using the same RNA that was utilized in the microarray analysis. To quantify nascent RNA, RT-PCR with real-time quantitation was performed using an iCycler system (Bio-Rad) as previously described [35]. RT-PCR primer sets for *ESR1*, *XBP1*, *CDH1*, *OCNL*, *DSP*, *GATA3*, *TGFB2*, *TGFBR2*, *FN1* and *18S* were obtained from Qiagen (Quantitect primers). Primer DNA sequences for *TAGLN*, *SPARC*, *CTGF* and *CLDN7* are listed in Table S2.

### Immunoblotting

Antibodies utilized included Snail (Abcam; ab17732) and Actin (Chemicon; MAB1501). Immunoblotting was performed using standard protocols.

### Chromatin Immunoprecipitation Analysis (ChIP)

Chromatin Immunoprecipitation was performed as previously described (Dhasarathy et al., 2007) using antibodies against Histone H3K9 acetyl (Upstate 07-352) and Histone H3K9 trimethyl (Abcam ab8898-100). Precipitated DNAs were detected by real time PCR using specific primers for the *TGFBR2* promoter (Table S2).

### Cell migration assay

The inhibitors were obtained from Sigma. Boyden chambers were prepared with the lower chamber containing DMEM/F-12 media with 10% fetal bovine serum (FBS) as chemoattractant and overlaid with nucleopore track-etch membranes (Whatman, 13 mm diameter, 8 µm pore size) coated with Human Collagen IV (BD Biosciences, 354245). MCF-7 cells treated with or without adenovirus and with or without inhibitors [SB431542 (Sigma, S4317) or LY364947 (Sigma, L6293)] or DMSO for 2 days, were resuspended in media without FBS and 20,000 cells placed in the upper chamber on the coated membrane. After 24 hours of incubation in a 37°C CO_2_ incubator, the non-migratory cells on the membrane surface were removed. The membranes (with migrated cells on the other side) were fixed with 4% formaldehyde, stained with 0.5% Crystal violet and mounted on glass slides. Ten fields were counted using a 5× lens on a Zeiss Axiovert 200 M microscope (Zeiss Microimaging Inc.).

## Supporting Information

Figure S1 **PCA analysis.** Principal Component Analysis (PCA) was performed on the background corrected and normalized data, for all probes and all samples as implemented in the programming language R (www.r-package.org). We used unsupervised hierarchical clustering of samples based on the differentially expressed probes at day 4 using the normalized data and average linkage clustering. This analysis revealed clear separation between the Snail and Slug treated MCF-7 samples relative to control. PC#1 = first principal component, PC#2 = second principal component, PC#3 = third principal component.
(TIF)

Figure S2 **Snail and Slug do not influence the expression of each other in MCF-7 cells.** The expression of Snail (A) and Slug (B) was examined in MCF-7 cells 2 and 4 days after addition of Snail or Slug adenovirus, using RT-PCR of cDNA with real-time quantitation following normalization to GAPDH, MCF-7 Day 0 and control adenovirus. The data represents the average of three independent biological replicates.
(TIF)

Figure S3 **RT-PCR validation of genes that are uniquely regulated by Snail and Slug.** cDNA was prepared from the same RNA that was used in the microarray experiment, and RT-PCR with real-time quantitation was performed using an iCycler system (Bio-Rad) to measure the RNA fold change following normalization to 18 s, MCF-7 Day 0 and control adenovirus. (A) RT-PCR validation of genes that change in Snail- but not Slug-expressing cells and (B) RT-PCR validation of genes that change in Slug- but not Snail-expressing cells.
(TIF)

Figure S4 **GSEA analysis following Snail and Slug expression.** GSEA analysis comparing the genes that were upregulated (A, C and E) or downregulated (B, D and F) in our microarray samples to those from the normal breast class (A and B), Her2-positive (C and D) and basal (E and F) categories of breast tumors described in [24].
(TIF)

Figure S5 **Relative RNA levels of TGF-beta markers in MCF-7 and MDA-MB-231 cells.** Relative to MCF-7, the highly invasive cell line MDA-MB-231 shows increased expression of FN1, TGFB2, TGFBR2, CTGF and SPARC, but not TGFB1. RNA was isolated from MCF-7 and MDA-MB-231 cells, cDNA was prepared and RT-PCR with real-time quantitation was performed using an iCycler system (Bio-Rad) to measure the RNA fold change following normalization to 18 s. The data represents the average of three independent biological replicates.
(TIF)

File S1 **IPA analysis.** We obtained the genes list for each of the following comparisons using the criteria: absolute (fold)>2 and adjusted pvalue<0.01: Snail Day 1 Vs. Mock Day 1, Snail Day 2 Vs. Mock Day 2, Snail Day 4 Vs. Mock Day 4, Slug Day 1 Vs. Mock Day 1, Slug Day 2 Vs. Mock Day 2 and Slug Day 4 Vs. Mock Day 4. We used the gene list described above as in input to find significantly enriched canonical pathways for each of the six comparisons by employing Ingenuity Pathway Analysis (IPA) software (www.ingenuity.com). Genes that are associated with a canonical pathway in the Ingenuity Knowledge Base were considered for the analysis. The significance of the association between the data set and the canonical pathway was measured in 2 ways: 1) A ratio of the number of genes from the data set that map to the pathway divided by the total number of genes that map to the canonical pathway, and 2) Fisher's exact test was used to calculate a p-value determining the probability that the association between the genes in the dataset and the canonical pathway is explained by chance alone (File S1).
(PDF)

Table S1 **Microarray genes changing over time with fold change>2, p-value<0.01 (adjusted), following Snail and Slug expression.** Probes that displayed a fold change of two-fold or greater in either direction, along with adjusted p-values less than 0.01 following Snail and Slug expression.
(PDF)

Table S2 **List of primers used in study.**
(PDF)
