# Supplementary figures and images for: The Transcription Factors Snail and Slug Activate the Transforming Growth Factor-Beta Signaling Pathway in Breast Cancer
Source: PLoS One. 2011 Oct 20;6(10):e26514. doi: 10.1371/journal.pone.0026514 (PMC3197668; doi:10.1371/journal.pone.0026514)

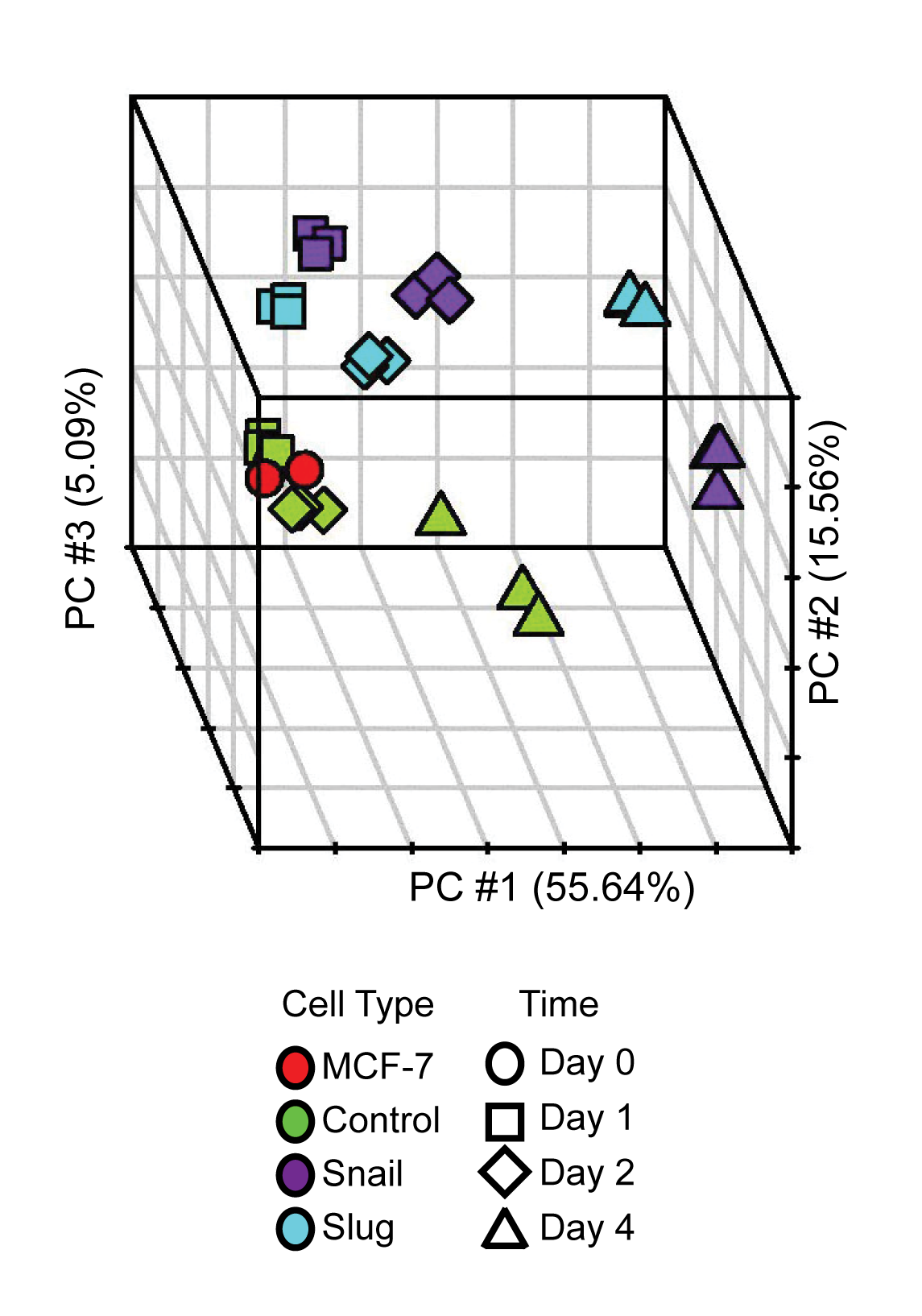

Supplement: Figure S1 — PCA analysis. Principal Component Analysis (PCA) was performed on the background corrected and normalized data, for all probes and all samples as implemented in the programming language R (www.r-package.org). We used unsupervised hierarchical clustering of samples based on the differentially expressed probes at day 4 using the normalized data and average linkage clustering. This analysis revealed clear separation between the Snail and Slug treated MCF-7 samples relative to control. PC#1 = first principal component, PC#2 = second principal component, PC#3 = third principal component. (TIF) [file pone.0026514.s001.tif]

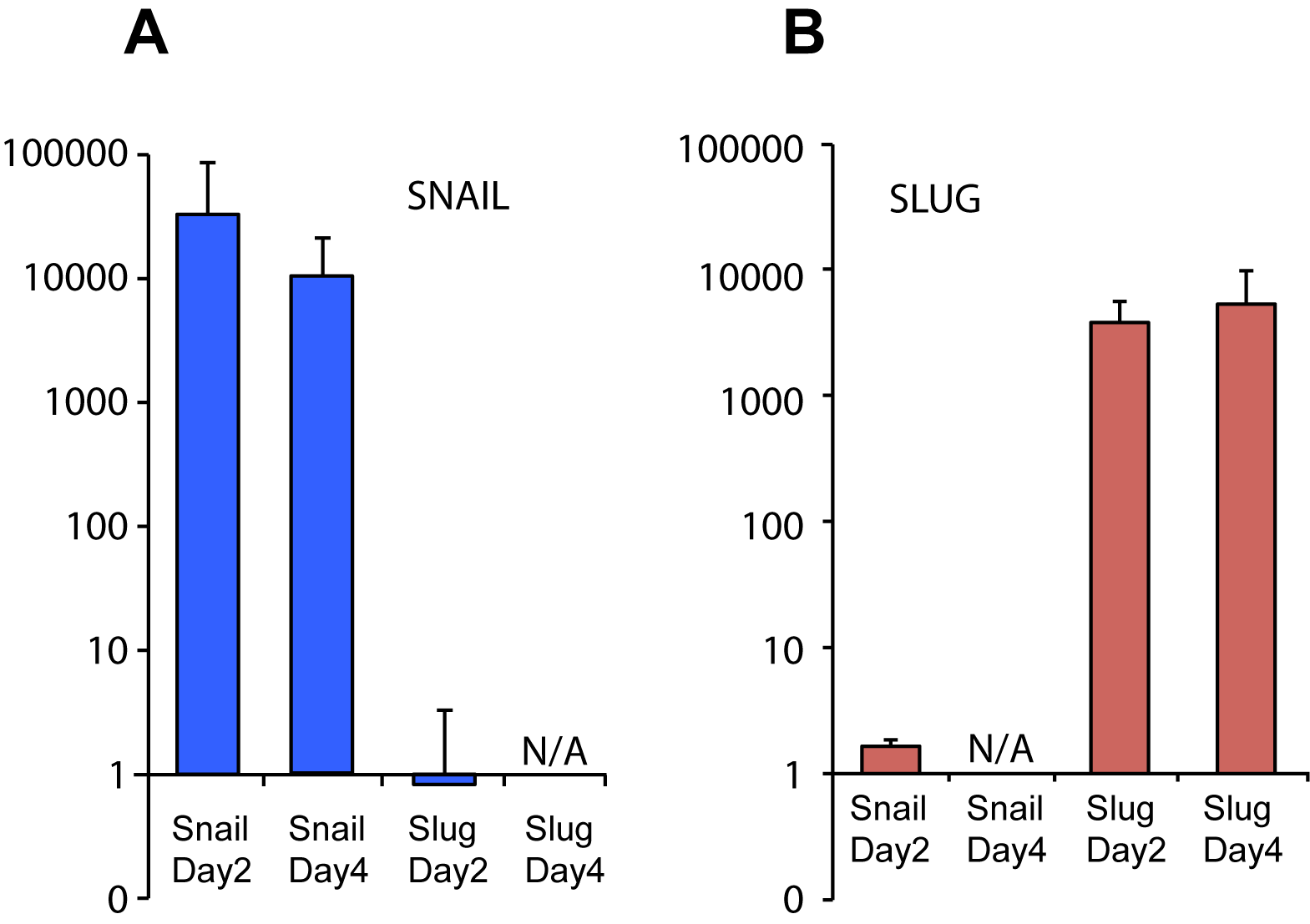

Supplement: Figure S2 — Snail and Slug do not influence the expression of each other in MCF-7 cells. The expression of Snail (A) and Slug (B) was examined in MCF-7 cells 2 and 4 days after addition of Snail or Slug adenovirus, using RT-PCR of cDNA with real-time quantitation following normalization to GAPDH, MCF-7 Day 0 and control adenovirus. The data represents the average of three independent biological replicates. (TIF) [file pone.0026514.s002.tif]

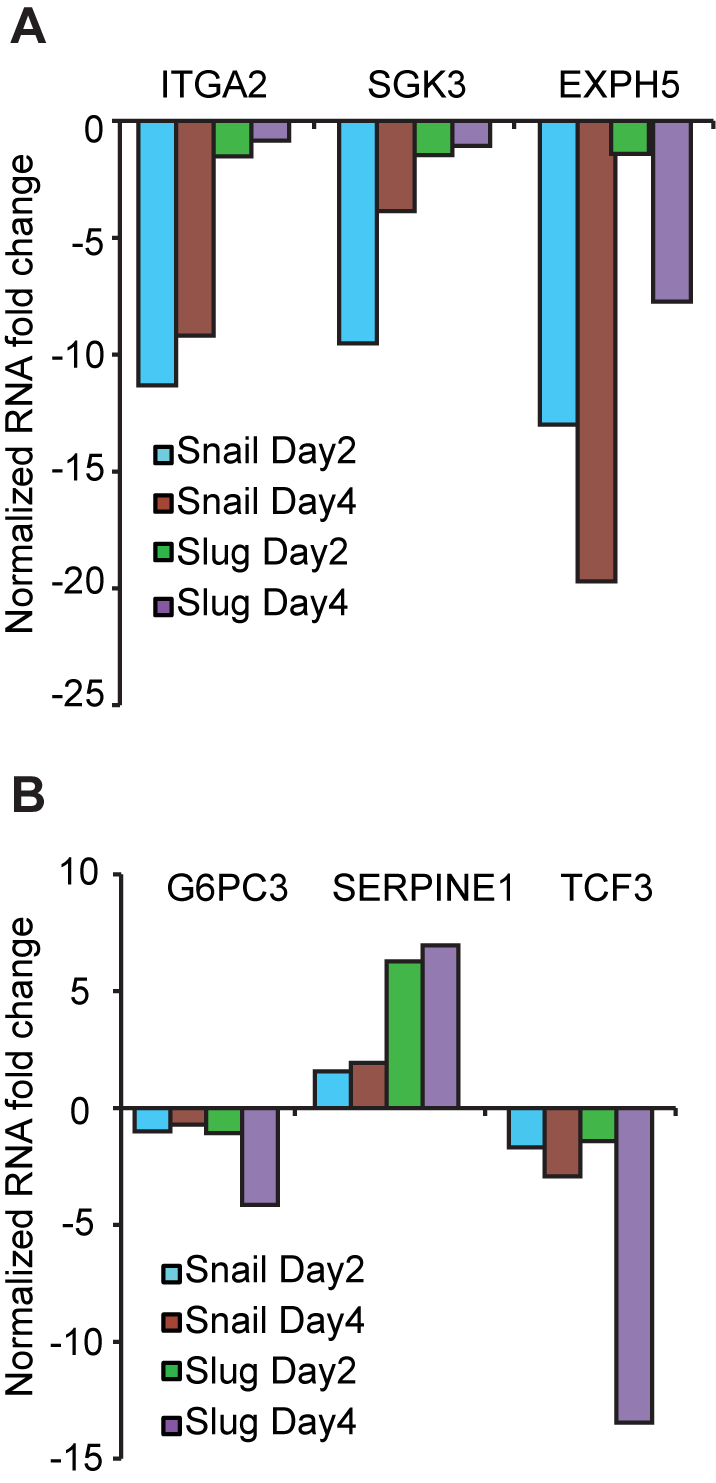

Supplement: Figure S3 — RT-PCR validation of genes that are uniquely regulated by Snail and Slug. cDNA was prepared from the same RNA that was used in the microarray experiment, and RT-PCR with real-time quantitation was performed using an iCycler system (Bio-Rad) to measure the RNA fold change following normalization to 18 s, MCF-7 Day 0 and control adenovirus. (A) RT-PCR validation of genes that change in Snail- but not Slug-expressing cells and (B) RT-PCR validation of genes that change in Slug- but not Snail-expressing cells. (TIF) [file pone.0026514.s003.tif]

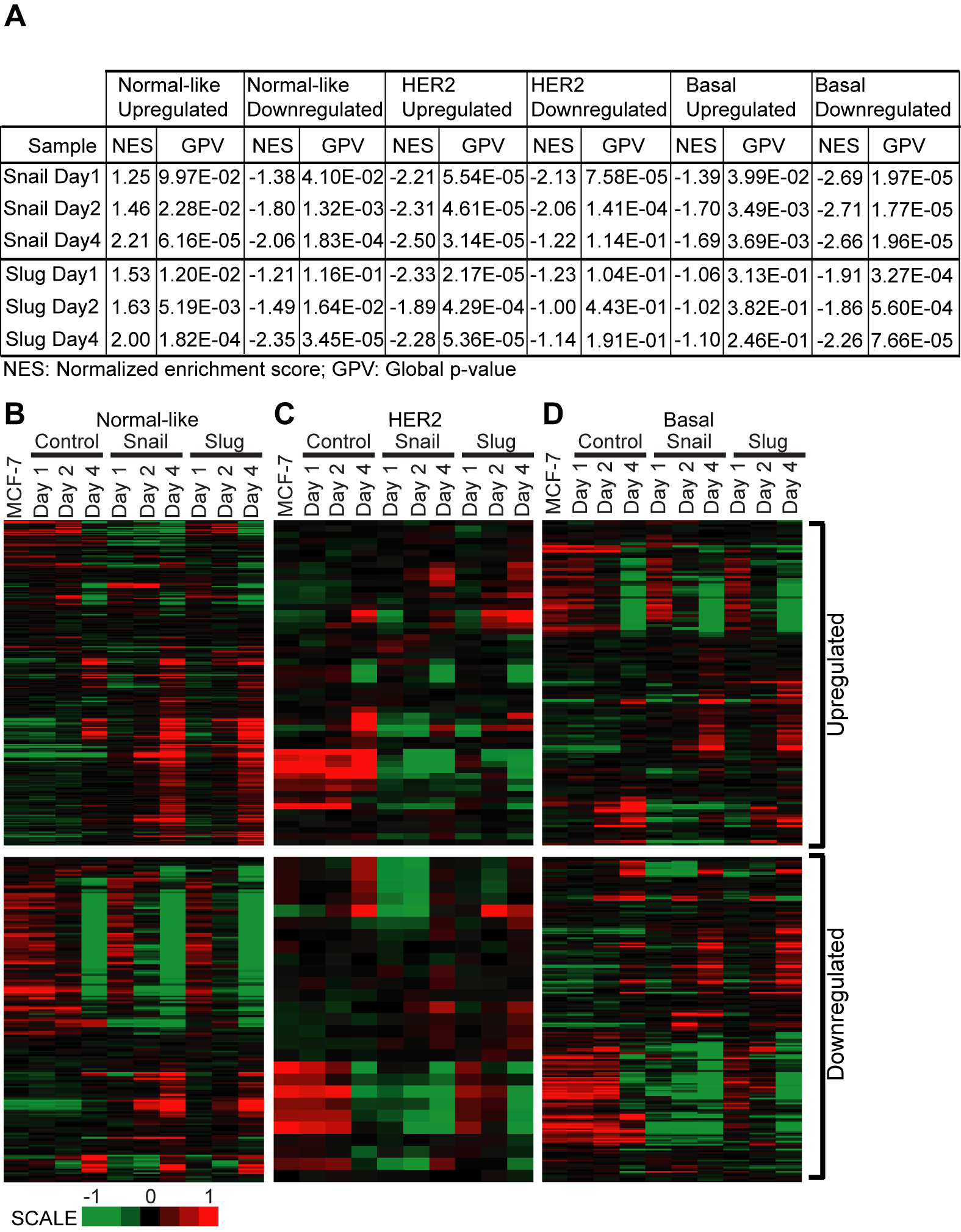

Supplement: Figure S4 — GSEA analysis following Snail and Slug expression. GSEA analysis comparing the genes that were upregulated (A, C and E) or downregulated (B, D and F) in our microarray samples to those from the normal breast class (A and B), Her2-positive (C and D) and basal (E and F) categories of breast tumors described in [24]. (TIF) [file pone.0026514.s004.tif]

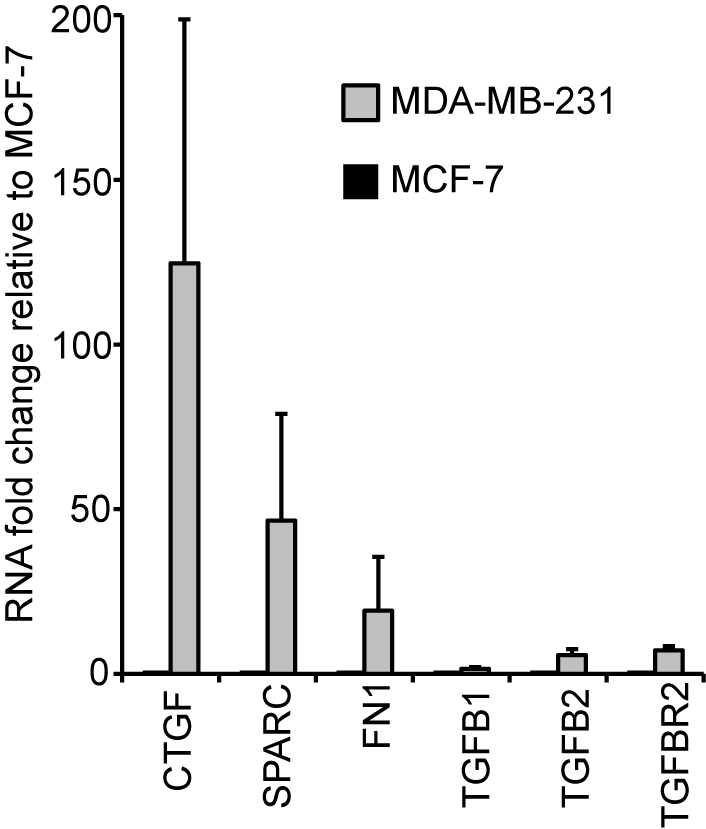

Supplement: Figure S5 — Relative RNA levels of TGF-beta markers in MCF-7 and MDA-MB-231 cells. Relative to MCF-7, the highly invasive cell line MDA-MB-231 shows increased expression of FN1, TGFB2, TGFBR2, CTGF and SPARC, but not TGFB1. RNA was isolated from MCF-7 and MDA-MB-231 cells, cDNA was prepared and RT-PCR with real-time quantitation was performed using an iCycler system (Bio-Rad) to measure the RNA fold change following normalization to 18 s. The data represents the average of three independent biological replicates. (TIF) [file pone.0026514.s005.tif]
