# Supplementary material for: The Transcription Factors Snail and Slug Activate the Transforming Growth Factor-Beta Signaling Pathway in Breast Cancer
Source: PLoS One. 2011 Oct 20;6(10):e26514. doi: 10.1371/journal.pone.0026514 (PMC3197668; doi:10.1371/journal.pone.0026514)
Supplement: File S1 — IPA analysis. We obtained the genes list for each of the following comparisons using the criteria: absolute (fold)>2 and adjusted pvalue<0.01: Snail Day 1 Vs. Mock Day 1, Snail Day 2 Vs. Mock Day 2, Snail Day 4 Vs. Mock Day 4, Slug Day 1 Vs. Mock Day 1, Slug Day 2 Vs. Mock Day 2 and Slug Day 4 Vs. Mock Day 4. We used the gene list described above as in input to find significantly enriched canonical pathways for each of the six comparisons by employing Ingenuity Pathway Analysis (IPA) software (www.ingenuity.com). Genes that are associated with a canonical pathway in the Ingenuity Knowledge Base were considered for the analysis. The significance of the association between the data set and the canonical pathway was measured in 2 ways: 1) A ratio of the number of genes from the data set that map to the pathway divided by the total number of genes that map to the canonical pathway, and 2) Fisher's exact test was used to calculate a p-value determining the probability that the association between the genes in the dataset and the canonical pathway is explained by chance alone (File S1). (PDF) [file pone.0026514.s006.pdf]

# IPA® Summary of Analysis

Analysis Name: snail_day1_fold=2_adjpval=0.01
Analysis Creation Date: 2011-05-23
IPA version: 9.0 (Release Date: 2011-05-20)
Content version: 3210 (Release Date: 2011-05-17)

## Analysis settings

View

Reference set: Human Genome U133 Plus 2.0 Array

Relationship to include: Direct and Indirect

Includes Endogenous Chemicals

Optional Analyses: My Pathways My List

Filter Summary:

Consider only relationships where

(confidence = Experimentally Observed) AND

(data sources = BIND OR BIOGRID OR ClinicalTrials.gov OR Cognia OR DIP OR Gene Ontology (GO) OR GVK Biosciences OR Ingenuity Expert Findings OR Ingenuity ExpertAssist Findings OR INTACT OR Interactome studies OR Kyoto Encyclopedia of Genes and Genomes (KEGG) OR MINT OR MIPS OR Obesity Gene Map Database OR TarBase)

## Top Networks

| ID | Associated Network Functions | Score |
|---|---|---|
| 1 | Cell Death, Cellular Movement, Lipid Metabolism | 45 |
| 2 | Gastrointestinal Disease, Genetic Disorder, Metabolic Disease | 37 |
| 3 | Cellular Development, Cellular Growth and Proliferation, Cell-To-Cell Signaling and Interaction | 35 |

(c) 2000-2011 Ingenuity Systems, Inc. All rights reserved.

| | | |
|---|---|---|
| 4 | Connective Tissue Development and Function, Tissue Morphology, Lipid Metabolism | 33 |
| 5 | Cellular Assembly and Organization, Nutritional Disease, Hair and Skin Development and Function | 32 |

(c) 2000-2011 Ingenuity Systems, Inc. All rights reserved.

## Top Bio Functions

### Diseases and Disorders

| Name | p-value | # Molecules |
|---|---|---|
| Cancer | 2.20E-05 - 4.25E-02 | 210 |
| Gastrointestinal Disease | 3.15E-05 - 3.88E-02 | 230 |
| Genetic Disorder | 3.15E-05 - 4.25E-02 | 371 |
| Inflammatory Disease | 3.15E-05 - 4.14E-02 | 172 |
| Reproductive System Disease | 7.35E-05 - 4.19E-02 | 130 |

### Molecular and Cellular Functions

| Name | p-value | # Molecules |
|---|---|---|
| Cellular Movement | 1.46E-05 - 3.88E-02 | 93 |
| Cell Death | 2.54E-05 - 4.23E-02 | 88 |
| Cell Cycle | 7.64E-05 - 4.25E-02 | 14 |
| Cellular Growth and Proliferation | 1.43E-04 - 4.10E-02 | 92 |
| Cell-To-Cell Signaling and Interaction | 2.11E-04 - 4.25E-02 | 75 |

### Physiological System Development and Function

| Name | p-value | # Molecules |
|---|---|---|
| Tissue Development | 2.11E-04 - 3.67E-02 | 83 |
| Tumor Morphology | 7.17E-04 - 3.87E-02 | 28 |
| Hematological System Development and Function | 1.39E-03 - 4.25E-02 | 41 |
| Humoral Immune Response | 1.39E-03 - 1.39E-03 | 3 |
| Immune Cell Trafficking | 1.39E-03 - 4.25E-02 | 9 |

(c) 2000-2011 Ingenuity Systems, Inc. All rights reserved.

## Top Canonical Pathways

| Name | p-value | Ratio |
|---|---|---|
| Glycosphingolipid Biosynthesis - Globoseries | 1.09E-02 | 4/39 (0.103) |
| Biosynthesis of Steroids | 1.09E-02 | 4/121 (0.033) |
| Tight Junction Signaling | 1.16E-02 | 13/164 (0.079) |
| HER-2 Signaling in Breast Cancer | 1.4E-02 | 8/81 (0.099) |
| Glycosphingolipid Biosynthesis - Ganglioseries | 1.75E-02 | 4/56 (0.071) |

## Top Molecules

### Fold Change up-regulated

| Molecules | Exp. Value | Exp. Chart |
|---|---|---|
| CTGF | ↑8.050 | |
| HSPA6* | ↑7.150 | |
| IL11 | ↑6.600 | |
| ANXA1 | ↑6.370 | |
| LIX1L | ↑4.660 | |
| LOXL2 | ↑4.600 | |
| SERPINE2 | ↑4.260 | |
| GEM | ↑4.190 | |
| CDH11* | ↑4.020 | |
| DSE | ↑4.010 | |

### Fold Change down-regulated

(c) 2000-2011 Ingenuity Systems, Inc. All rights reserved.

| Molecules | Exp. Value | Exp. Chart |
|---|---|---|
| SLC6A14 | ↓21.810 | |
| PRR15L | ↓21.470 | |
| DOK7 | ↓17.190 | |
| CLDN3* | ↓13.720 | |
| SLC27A2* | ↓12.050 | |
| PLS1 | ↓11.410 | |
| GPR160 | ↓10.050 | |
| CYB5A* | ↓9.870 | |
| RAB25 | ↓8.340 | |
| AGPAT3* | ↓8.230 | |

(c) 2000-2011 Ingenuity Systems, Inc. All rights reserved.

INGENUITY SYSTEMS

## Top My Lists

| Name | p-value | Ratio |
|---|---|---|

## Top My Pathways

| Name | p-value | Ratio |
|---|---|---|

## Top Tox Lists

| Name | p-value | Ratio |
|---|---|---|
| Cytochrome P450 Panel - Substrate is a Vitamin (Human) | 1.66E-02 | 2/6 (0.333) |
| Cytochrome P450 Panel - Substrate is a Vitamin (Mouse) | 2.41E-02 | 2/7 (0.286) |
| Cytochrome P450 Panel - Substrate is a Vitamin (Rat) | 2.41E-02 | 2/7 (0.286) |
| Liver Proliferation | 1.06E-01 | 9/133 (0.068) |
| p53 Signaling | 1.09E-01 | 7/95 (0.074) |

(c) 2000-2011 Ingenuity Systems, Inc. All rights reserved.

INGENUITY SYSTEMS

## Top Tox Functions

### Assays: Clinical Chemistry and Hematology

| Name | p-value | # Molecules |
| --- | --- | --- |
| Increased Levels of Potassium | 8.32E-02 - 8.32E-02 | 1 |
| Increased Levels of AST | 2.29E-01 - 2.29E-01 | 1 |
| Increased Levels of Alkaline Phosphatase | 4.85E-01 - 4.85E-01 | 2 |

### Cardiotoxicity

| Name | p-value | # Molecules |
| --- | --- | --- |
| Cardiac Arteriopathy | 6.78E-02 - 2.62E-01 | 66 |
| Cardiac Arrythmia | 8.32E-02 - 1.00E00 | 4 |
| Cardiac Damage | 8.32E-02 - 4.32E-01 | 2 |
| Heart Failure | 8.32E-02 - 1.00E00 | 8 |
| Cardiac Dysfunction | 1.60E-01 - 5.43E-01 | 4 |

### Hepatotoxicity

| Name | p-value | # Molecules |
| --- | --- | --- |
| Liver Adhesion | 5.26E-03 - 5.26E-03 | 2 |
| Liver Cirrhosis | 6.52E-03 - 1.09E-01 | 11 |
| Liver Hemorrhaging | 1.02E-02 - 1.02E-02 | 2 |
| Liver Cholestasis | 4.25E-02 - 1.95E-01 | 8 |
| Liver Hepatitis | 4.26E-02 - 1.00E00 | 6 |

### Nephrotoxicity

(c) 2000-2011 Ingenuity Systems, Inc. All rights reserved.

| Name | p-value | # Molecules |
|---|---|---|
| Renal Dysplasia | 1.02E-02 - 4.25E-02 | 2 |
| Renal Hypoplasia | 4.25E-02 - 4.25E-02 | 1 |
| Renal Tubule Injury | 4.25E-02 - 5.62E-01 | 8 |
| Renal Nephritis | 6.02E-02 - 3.24E-01 | 8 |
| Renal Enlargement | 8.32E-02 - 8.32E-02 | 1 |

(c) 2000-2011 Ingenuity Systems, Inc. All rights reserved.

INGENUITY® SYSTEMS

# IPA® Summary of Analysis

Analysis Name: snail_day2_fold=2_adjpval=0.01
Analysis Creation Date: 2011-05-23
IPA version: 9.0 (Release Date: 2011-05-20)
Content version: 3210 (Release Date: 2011-05-17)

## Analysis settings

View

Reference set: Human Genome U133 Plus 2.0 Array

Relationship to include: Direct and Indirect

Includes Endogenous Chemicals

Optional Analyses: My Pathways My List

Filter Summary:

Consider only relationships where

(confidence = Experimentally Observed) AND

(data sources = BIND OR BIOGRID OR ClinicalTrials.gov OR Cognia OR DIP OR Gene Ontology (GO) OR GVK Biosciences OR Ingenuity Expert Findings OR Ingenuity ExpertAssist Findings OR INTACT OR Interactome studies OR Kyoto Encyclopedia of Genes and Genomes (KEGG) OR MINT OR MIPS OR Obesity Gene Map Database OR TarBase)

## Top Networks

| ID | Associated Network Functions | Score |
|---|---|---|
| 1 | Amino Acid Metabolism, Small Molecule Biochemistry, Genetic Disorder | 45 |
| 2 | Cellular Assembly and Organization, Cell Death, Cell-To-Cell Signaling and Interaction | 40 |
| 3 | Cellular Development, Cellular Growth and Proliferation, Nervous System Development and Function | 36 |

(c) 2000-2011 Ingenuity Systems, Inc. All rights reserved.

| | | |
|---|---|---|
| 4 | Cellular Assembly and Organization, Drug Metabolism, Genetic Disorder | 34 |
| 5 | Cell Signaling, Cellular Function and Maintenance, Molecular Transport | 34 |

(c) 2000-2011 Ingenuity Systems, Inc. All rights reserved.

## Top Bio Functions

### Diseases and Disorders

| Name | p-value | # Molecules |
|---|---|---|
| Cancer | 2.42E-12 - 1.47E-02 | 369 |
| Reproductive System Disease | 1.87E-07 - 1.29E-02 | 225 |
| Gastrointestinal Disease | 2.29E-07 - 1.31E-02 | 384 |
| Genetic Disorder | 5.37E-07 - 1.47E-02 | 632 |
| Hepatic System Disease | 8.19E-06 - 1.31E-02 | 68 |

### Molecular and Cellular Functions

| Name | p-value | # Molecules |
|---|---|---|
| Cell Death | 4.88E-09 - 1.57E-02 | 282 |
| Cellular Movement | 1.90E-08 - 1.51E-02 | 123 |
| Cellular Growth and Proliferation | 7.87E-05 - 1.53E-02 | 285 |
| Cell-To-Cell Signaling and Interaction | 1.24E-04 - 1.51E-02 | 96 |
| Cellular Compromise | 1.24E-04 - 1.47E-02 | 16 |

### Physiological System Development and Function

| Name | p-value | # Molecules |
|---|---|---|
| Hematological System Development and Function | 5.15E-05 - 1.47E-02 | 71 |
| Tissue Development | 1.24E-04 - 1.57E-02 | 111 |
| Tumor Morphology | 1.24E-04 - 1.47E-02 | 43 |
| Hair and Skin Development and Function | 2.41E-04 - 1.53E-02 | 48 |
| Cardiovascular System Development and Function | 3.68E-04 - 1.47E-02 | 41 |

(c) 2000-2011 Ingenuity Systems, Inc. All rights reserved.

## Top Canonical Pathways

| Name | p-value | Ratio |
|---|---|---|
| Glycosphingolipid Biosynthesis - Globoseries | 2.86E-03 | 6/39 (0.154) |
| Biosynthesis of Steroids | 2.86E-03 | 6/121 (0.05) |
| Mechanisms of Viral Exit from Host Cells | 6.68E-03 | 8/45 (0.178) |
| HER-2 Signaling in Breast Cancer | 6.8E-03 | 12/81 (0.148) |
| Glycosphingolipid Biosynthesis - Lactoseries | 8.17E-03 | 4/27 (0.148) |

## Top Molecules

### Fold Change up-regulated

| Molecules | Exp. Value | Exp. Chart |
|---|---|---|
| IL11 | ↑10.300 | |
| LIX1L | ↑8.230 | |
| CTGF | ↑7.870 | |
| SERPINE2 | ↑7.810 | |
| EDIL3 | ↑7.430 | |
| TGFBI | ↑7.110 | |
| LOXL2* | ↑6.940 | |
| CDH11* | ↑6.880 | |
| TAGLN* | ↑6.520 | |
| IKBIP* | ↑6.080 | |

### Fold Change down-regulated

(c) 2000-2011 Ingenuity Systems, Inc. All rights reserved.

| Molecules | Exp. Value | Exp. Chart |
|---|---|---|
| SLC6A14 | ↓60.460 | |
| GPR160 | ↓33.930 | |
| PRR15L | ↓26.750 | |
| CLDN3* | ↓22.250 | |
| ITGB6 | ↓20.870 | |
| PRLR* | ↓16.010 | |
| PLS1 | ↓15.820 | |
| ANXA9* | ↓15.470 | |
| CYB5A* | ↓15.130 | |
| RAB25 | ↓15.070 | |

(c) 2000-2011 Ingenuity Systems, Inc. All rights reserved.

INGENUITY® SYSTEMS

## Top My Lists

| Name | p-value | Ratio |
|---|---|---|

## Top My Pathways

| Name | p-value | Ratio |
|---|---|---|

## Top Tox Lists

| Name | p-value | Ratio |
|---|---|---|
| Liver Proliferation | 1.24E-02 | 17/133 (0.128) |
| Increases Permeability Transition of Mitochondria and Mitochondrial Membrane | 1.57E-02 | 3/8 (0.375) |
| Increases Transmembrane Potential of Mitochondria and Mitochondrial Membrane | 1.98E-02 | 8/50 (0.16) |
| Decreases Depolarization of Mitochondria and Mitochondrial Membrane | 2.23E-02 | 3/9 (0.333) |
| Pro-Apoptosis | 2.84E-02 | 7/42 (0.167) |

(c) 2000-2011 Ingenuity Systems, Inc. All rights reserved.

## Top Tox Functions

### Assays: Clinical Chemistry and Hematology

| Name | p-value | # Molecules |
|---|---|---|
| Increased Levels of ALT | 4.44E-02 - 4.44E-02 | 2 |
| Increased Levels of LDH | 1.32E-01 - 1.32E-01 | 2 |
| Increased Levels of Potassium | 1.38E-01 - 1.38E-01 | 1 |
| Increased Levels of AST | 3.60E-01 - 3.60E-01 | 1 |
| Increased Levels of Alkaline Phosphatase | 5.20E-01 - 5.20E-01 | 3 |

### Cardiotoxicity

| Name | p-value | # Molecules |
|---|---|---|
| Cardiac Arteriopathy | 4.66E-03 - 4.06E-01 | 117 |
| Cardiac Arrythmia | 6.35E-02 - 6.20E-01 | 4 |
| Pulmonary Hypertension | 1.09E-01 - 2.00E-01 | 6 |
| Cardiac Damage | 1.38E-01 - 5.22E-01 | 2 |
| Cardiac Enlargement | 1.38E-01 - 1.38E-01 | 1 |

### Hepatotoxicity

| Name | p-value | # Molecules |
|---|---|---|
| Liver Cirrhosis | 9.88E-04 - 3.58E-02 | 19 |
| Liver Degeneration | 3.89E-03 - 3.89E-03 | 4 |
| Liver Adhesion | 1.47E-02 - 1.47E-02 | 2 |
| Liver Hematopoiesis | 1.47E-02 - 1.47E-02 | 2 |
| Liver Necrosis/Cell Death | 1.53E-02 - 6.20E-01 | 16 |

(c) 2000-2011 Ingenuity Systems, Inc. All rights reserved.

INGENUITY SYSTEMS

**Nephrotoxicity**

| Name | p-value | # Molecules |
|---|---|---|
| Renal Dysplasia | 2.63E-05 - 7.17E-02 | 4 |
| Renal Damage | 2.80E-02 - 4.49E-01 | 3 |
| Renal Tubule Injury | 2.80E-02 - 7.17E-02 | 13 |
| Renal Necrosis/Cell Death | 3.21E-02 - 5.87E-01 | 31 |
| Glomerular Injury | 7.17E-02 - 4.26E-01 | 3 |

(c) 2000-2011 Ingenuity Systems, Inc. All rights reserved.

# IPA® Summary of Analysis

Analysis Name: snail_day4_fold=2_adjpval=0.01
Analysis Creation Date: 2011-05-23
IPA version: 9.0 (Release Date: 2011-05-20)
Content version: 3210 (Release Date: 2011-05-17)

## Analysis settings

View

Reference set: Human Genome U133 Plus 2.0 Array

Relationship to include: Direct and Indirect

Includes Endogenous Chemicals

Optional Analyses: My Pathways My List

Filter Summary:

Consider only relationships where

(confidence = Experimentally Observed) AND

(data sources = BIND OR BIOGRID OR ClinicalTrials.gov OR Cognia OR DIP OR Gene Ontology (GO) OR GVK Biosciences OR Ingenuity Expert Findings OR Ingenuity ExpertAssist Findings OR INTACT OR Interactome studies OR Kyoto Encyclopedia of Genes and Genomes (KEGG) OR MINT OR MIPS OR Obesity Gene Map Database OR TarBase)

## Top Networks

| ID | Associated Network Functions | Score |
|---|---|---|
| 1 | Amino Acid Metabolism, Cellular Assembly and Organization, Gastrointestinal Disease | 48 |
| 2 | Post-Translational Modification, Molecular Transport, Genetic Disorder | 38 |
| 3 | Cellular Assembly and Organization, Gene Expression, Cell Cycle | 36 |

(c) 2000-2011 Ingenuity Systems, Inc. All rights reserved.

| | | |
|---|---|---|
| 4 | Inflammatory Disease, Respiratory Disease, Auditory Disease | 36 |
| 5 | Cell Cycle, Cancer, Reproductive System Disease | 36 |

(c) 2000-2011 Ingenuity Systems, Inc. All rights reserved.

## Top Bio Functions

### Diseases and Disorders

| Name | p-value | # Molecules |
|---|---|---|
| Cancer | 4.80E-18 - 5.14E-03 | 386 |
| Gastrointestinal Disease | 4.99E-15 - 5.10E-03 | 408 |
| Genetic Disorder | 1.70E-10 - 4.79E-03 | 629 |
| Reproductive System Disease | 2.56E-10 - 3.42E-03 | 234 |
| Dermatological Diseases and Conditions | 1.31E-07 - 5.64E-03 | 150 |

### Molecular and Cellular Functions

| Name | p-value | # Molecules |
|---|---|---|
| Cellular Movement | 1.18E-13 - 5.64E-03 | 187 |
| Cell Death | 1.29E-08 - 5.40E-03 | 282 |
| Cell-To-Cell Signaling and Interaction | 4.26E-08 - 5.64E-03 | 123 |
| Cellular Growth and Proliferation | 2.54E-06 - 4.95E-03 | 288 |
| Cellular Development | 2.88E-06 - 5.64E-03 | 221 |

### Physiological System Development and Function

| Name | p-value | # Molecules |
|---|---|---|
| Tissue Development | 4.26E-08 - 5.64E-03 | 127 |
| Organ Development | 9.67E-07 - 4.79E-03 | 107 |
| Reproductive System Development and Function | 9.67E-07 - 5.08E-03 | 26 |
| Organismal Functions | 2.24E-05 - 1.27E-04 | 20 |
| Endocrine System Development and Function | 2.28E-05 - 5.64E-03 | 16 |

(c) 2000-2011 Ingenuity Systems, Inc. All rights reserved.

## Top Canonical Pathways

| Name | p-value | Ratio |
|---|---|---|
| Aryl Hydrocarbon Receptor Signaling | 3.02E-05 | 23/159 (0.145) |
| Mechanisms of Viral Exit from Host Cells | 5.92E-05 | 11/45 (0.244) |
| NRF2-mediated Oxidative Stress Response | 7.62E-05 | 27/193 (0.14) |
| Antiproliferative Role of TOB in T Cell Signaling | 2.6E-04 | 8/26 (0.308) |
| Glutathione Metabolism | 9.95E-04 | 10/90 (0.111) |

## Top Molecules

### Fold Change up-regulated

| Molecules | Exp. Value | Exp. Chart |
|---|---|---|
| CTGF | ↑27.310 | |
| SRPX | ↑26.010 | |
| IGFBP7* | ↑24.020 | |
| TAGLN* | ↑17.350 | |
| CALD1* | ↑16.790 | |
| EDIL3 | ↑14.510 | |
| COL6A1* | ↑13.940 | |
| LOXL2* | ↑13.590 | |
| FHL1* | ↑12.430 | |
| COL5A1* | ↑9.210 | |

### Fold Change down-regulated

(c) 2000-2011 Ingenuity Systems, Inc. All rights reserved.

| Molecules | Exp. Value | Exp. Chart |
|---|---|---|
| GPR160 | ↓44.160 | |
| AQP3* | ↓43.180 | |
| SLC6A14 | ↓36.950 | |
| CXCR7 | ↓31.960 | |
| PKIB* | ↓28.250 | |
| ITGB6* | ↓27.960 | |
| CEACAM1 (includes others)* | ↓23.720 | |
| GRHL1* | ↓20.400 | |
| ZNF165 | ↓19.450 | |
| CLDN3* | ↓16.950 | |

(c) 2000-2011 Ingenuity Systems, Inc. All rights reserved.

## Top My Lists

| Name | p-value | Ratio |
|---|---|---|

## Top My Pathways

| Name | p-value | Ratio |
|---|---|---|

## Top Tox Lists

| Name | p-value | Ratio |
|---|---|---|
| Oxidative Stress | 1.25E-05 | 14/57 (0.246) |
| Hepatic Fibrosis | 5.15E-05 | 17/85 (0.2) |
| Glutathione Depletion - Phase II Reactions | 7.83E-05 | 7/21 (0.333) |
| Aryl Hydrocarbon Receptor Signaling | 1.1E-04 | 23/157 (0.146) |
| NRF2-mediated Oxidative Stress Response | 3.3E-04 | 28/237 (0.118) |

(c) 2000-2011 Ingenuity Systems, Inc. All rights reserved.

## Top Tox Functions

### Assays: Clinical Chemistry and Hematology

| Name | p-value | # Molecules |
|---|---|---|
| Increased Levels of Albumin | 6.92E-02 - 6.92E-02 | 1 |
| Increased Levels of Alkaline Phosphatase | 6.92E-02 - 1.19E-01 | 6 |
| Decreased Levels of Albumin | 1.94E-01 - 3.50E-01 | 2 |
| Increased Levels of Red Blood Cells | 1.94E-01 - 1.94E-01 | 1 |
| Increased Levels of ALT | 3.01E-01 - 3.01E-01 | 1 |

### Cardiotoxicity

| Name | p-value | # Molecules |
|---|---|---|
| Cardiac Hypertrophy | 2.63E-03 - 5.46E-01 | 24 |
| Cardiac Dilation | 3.02E-02 - 3.99E-02 | 7 |
| Cardiac Arrythmia | 5.96E-02 - 1.00E00 | 11 |
| Cardiac Enlargement | 6.92E-02 - 2.49E-01 | 3 |
| Cardiac Necrosis/Cell Death | 6.92E-02 - 4.02E-01 | 13 |

### Hepatotoxicity

| Name | p-value | # Molecules |
|---|---|---|
| Liver Cirrhosis | 1.08E-04 - 1.94E-01 | 26 |
| Liver Adhesion | 1.57E-04 - 1.37E-02 | 5 |
| Glutathione Depletion In Liver | 4.75E-04 - 6.92E-02 | 8 |
| Liver Fibrosis | 1.21E-03 - 4.76E-01 | 17 |
| Hepatocellular Carcinoma | 1.25E-03 - 1.25E-03 | 27 |

(c) 2000-2011 Ingenuity Systems, Inc. All rights reserved.

INGENUITY® SYSTEMS

## Nephrotoxicity

| Name | p-value | # Molecules |
| --- | --- | --- |
| Renal Damage | 1.25E-03 - 1.00E00 | 8 |
| Renal Tubule Injury | 1.25E-03 - 3.83E-01 | 16 |
| Renal Necrosis/Cell Death | 2.63E-03 - 1.00E00 | 33 |
| Kidney Failure | 1.20E-02 - 3.01E-01 | 15 |
| Renal Dysplasia | 2.62E-02 - 6.92E-02 | 2 |

(c) 2000-2011 Ingenuity Systems, Inc. All rights reserved.

INGENUITY® SYSTEMS

# IPA® Summary of Analysis

Analysis Name: slug_day1_fold=2_adjpval=0.01
Analysis Creation Date: 2011-05-23
IPA version: 9.0 (Release Date: 2011-05-20)
Content version: 3210 (Release Date: 2011-05-17)

## Analysis settings

View

Reference set: Human Genome U133 Plus 2.0 Array

Relationship to include: Direct and Indirect

Includes Endogenous Chemicals

Optional Analyses: My Pathways My List

Filter Summary:

Consider only relationships where

(confidence = Experimentally Observed) AND

(data sources = BIND OR BIOGRID OR ClinicalTrials.gov OR Cognia OR DIP OR Gene Ontology (GO) OR GVK Biosciences OR Ingenuity Expert Findings OR Ingenuity ExpertAssist Findings OR INTACT OR Interactome studies OR Kyoto Encyclopedia of Genes and Genomes (KEGG) OR MINT OR MIPS OR Obesity Gene Map Database OR TarBase)

## Top Networks

| ID | Associated Network Functions | Score |
|---|---|---|
| 1 | Cellular Development, Cell Cycle, Cancer | 33 |
| 2 | Cell-To-Cell Signaling and Interaction, Tissue Development, Connective Tissue Development and Function | 32 |
| 3 | Cellular Function and Maintenance, Cell-To-Cell Signaling and Interaction, Hematological System Development and Function | 29 |

(c) 2000-2011 Ingenuity Systems, Inc. All rights reserved.

| | | |
|---|---|---|
| 4 | Skeletal and Muscular System Development and Function, Tissue Development, Carbohydrate Metabolism | 27 |
| 5 | Cell Morphology, Cellular Assembly and Organization, Nervous System Development and Function | 22 |

(c) 2000-2011 Ingenuity Systems, Inc. All rights reserved.

INGENUITY® SYSTEMS

## Top Bio Functions

### Diseases and Disorders

| Name | p-value | # Molecules |
|---|---|---|
| Dermatological Diseases and Conditions | 9.08E-06 - 1.10E-02 | 21 |
| Genetic Disorder | 9.08E-06 - 1.10E-02 | 54 |
| Cancer | 1.26E-05 - 1.10E-02 | 36 |
| Gastrointestinal Disease | 1.26E-05 - 1.10E-02 | 21 |
| Reproductive System Disease | 1.56E-05 - 9.54E-03 | 29 |

### Molecular and Cellular Functions

| Name | p-value | # Molecules |
|---|---|---|
| Cell Death | 4.09E-08 - 1.10E-02 | 28 |
| Cell-To-Cell Signaling and Interaction | 7.93E-08 - 1.10E-02 | 27 |
| Cellular Development | 5.16E-07 - 1.10E-02 | 32 |
| Cellular Growth and Proliferation | 5.20E-06 - 1.10E-02 | 32 |
| Cellular Movement | 1.90E-05 - 1.06E-02 | 24 |

### Physiological System Development and Function

| Name | p-value | # Molecules |
|---|---|---|
| Tissue Development | 7.93E-08 - 1.10E-02 | 30 |
| Connective Tissue Development and Function | 5.16E-07 - 1.10E-02 | 16 |
| Skeletal and Muscular System Development and Function | 1.69E-06 - 1.10E-02 | 14 |
| Hepatic System Development and Function | 9.04E-05 - 1.10E-02 | 3 |
| Organ Development | 1.08E-04 - 1.10E-02 | 18 |

(c) 2000-2011 Ingenuity Systems, Inc. All rights reserved.

INGENUITY® SYSTEMS

## Top Canonical Pathways

| Name | p-value | Ratio |
|---|---|---|
| Aldosterone Signaling in Epithelial Cells | 1.44E-03 | 5/170 (0.029) |
| p53 Signaling | 1.74E-03 | 4/96 (0.042) |
| Role of Tissue Factor in Cancer | 2.71E-03 | 4/114 (0.035) |
| Glucocorticoid Receptor Signaling | 2.91E-03 | 6/295 (0.02) |
| ERK5 Signaling | 4.45E-03 | 3/64 (0.047) |

## Top Molecules

### Fold Change up-regulated

| Molecules | Exp. Value | Exp. Chart |
|---|---|---|
| HSPA6* | ↑13.440 | |
| CTGF | ↑7.390 | |
| IL11 | ↑6.820 | |
| SERPINE2 | ↑5.100 | |
| LARP6 | ↑4.600 | |
| SERPINE1* | ↑4.390 | |
| FAS* | ↑3.640 | |
| FYN | ↑3.410 | |
| RGS2 | ↑3.400 | |
| TGFBI | ↑3.260 | |

### Fold Change down-regulated

(c) 2000-2011 Ingenuity Systems, Inc. All rights reserved.

| Molecules | Exp. Value | Exp. Chart |
|---|---|---|
| PRR15L | ↓4.850 | |
| SAMD12 | ↓3.360 | |
| CALML5 | ↓3.230 | |
| MACC1* | ↓2.950 | |
| SLC27A2* | ↓2.720 | |
| FOXC1 | ↓2.510 | |
| BAG1 | ↓2.460 | |
| CYFIP2 | ↓2.380 | |
| AQP3 | ↓2.350 | |
| FBP1 | ↓2.330 | |

(c) 2000-2011 Ingenuity Systems, Inc. All rights reserved.

INGENUITY® SYSTEMS

## Top My Lists

| Name | p-value | Ratio |
|---|---|---|

## Top My Pathways

| Name | p-value | Ratio |
|---|---|---|

## Top Tox Lists

| Name | p-value | Ratio |
|---|---|---|
| p53 Signaling | 1.88E-03 | 4/95 (0.042) |
| VDR/RXR Activation | 9.21E-03 | 3/78 (0.038) |
| Cardiac Hypertrophy | 1.3E-02 | 5/259 (0.019) |
| Hepatic Stellate Cell Activation | 1.6E-02 | 2/35 (0.057) |
| Renal Necrosis/Cell Death | 2.72E-02 | 5/314 (0.016) |

(c) 2000-2011 Ingenuity Systems, Inc. All rights reserved.

INGENUITY® SYSTEMS

## Top Tox Functions

### Assays: Clinical Chemistry and Hematology

| Name | p-value | # Molecules |
|---|---|---|
| Increased Levels of Alkaline Phosphatase | 1.20E-03 - 1.20E-03 | 3 |
| Increased Levels of Potassium | 1.10E-02 - 1.10E-02 | 1 |
| Increased Levels of AST | 3.28E-02 - 3.28E-02 | 1 |

### Cardiotoxicity

| Name | p-value | # Molecules |
|---|---|---|
| Cardiac Damage | 1.10E-02 - 2.20E-02 | 1 |
| Heart Failure | 1.10E-02 - 2.80E-01 | 3 |
| Cardiac Hypertrophy | 1.70E-02 - 1.63E-01 | 6 |
| Cardiac Dysfunction | 9.01E-02 - 9.01E-02 | 1 |
| Cardiac Fibrosis | 9.51E-02 - 1.99E-01 | 1 |

### Hepatotoxicity

| Name | p-value | # Molecules |
|---|---|---|
| Liver Adhesion | 9.04E-05 - 9.04E-05 | 2 |
| Liver Cirrhosis | 1.74E-03 - 1.74E-03 | 4 |
| Liver Damage | 1.10E-02 - 6.33E-02 | 2 |
| Liver Necrosis/Cell Death | 1.10E-02 - 7.72E-02 | 2 |
| Liver Regeneration | 1.10E-02 - 5.40E-02 | 2 |

### Nephrotoxicity

(c) 2000-2011 Ingenuity Systems, Inc. All rights reserved.

| Name | p-value | # Molecules |
|---|---|---|
| Renal Nephritis | 1.93E-03 - 4.87E-02 | 3 |
| Renal Necrosis/Cell Death | 3.28E-02 - 9.36E-02 | 5 |
| Renal Tubule Injury | 7.41E-02 - 7.41E-02 | 2 |
| Kidney Failure | 1.58E-01 - 1.58E-01 | 1 |
| Renal Proliferation | 1.72E-01 - 1.72E-01 | 1 |

(c) 2000-2011 Ingenuity Systems, Inc. All rights reserved.

# IPA® Summary of Analysis

Analysis Name: slug_day2_fold=2_adjpval=0.01
Analysis Creation Date: 2011-05-23
IPA version: 9.0 (Release Date: 2011-05-20)
Content version: 3210 (Release Date: 2011-05-17)

## Analysis settings

View

Reference set: Human Genome U133 Plus 2.0 Array

Relationship to include: Direct and Indirect

Includes Endogenous Chemicals

Optional Analyses: My Pathways My List

Filter Summary:

Consider only relationships where

(confidence = Experimentally Observed) AND

(data sources = BIND OR BIOGRID OR ClinicalTrials.gov OR Cognia OR DIP OR Gene Ontology (GO) OR GVK Biosciences OR Ingenuity Expert Findings OR Ingenuity ExpertAssist Findings OR INTACT OR Interactome studies OR Kyoto Encyclopedia of Genes and Genomes (KEGG) OR MINT OR MIPS OR Obesity Gene Map Database OR TarBase)

## Top Networks

| ID | Associated Network Functions | Score |
|---|---|---|
| 1 | Cellular Compromise, Nutritional Disease, Cell Death | 36 |
| 2 | Cell-To-Cell Signaling and Interaction, Tissue Development, Cardiovascular System Development and Function | 27 |
| 3 | Cell Cycle, Cell Death, Cellular Development | 22 |

(c) 2000-2011 Ingenuity Systems, Inc. All rights reserved.

| | | |
|---|---|---|
| 4 | Cellular Assembly and Organization, Cellular Movement, Neurological Disease | 22 |
| 5 | Metabolic Disease, Carbohydrate Metabolism, Lipid Metabolism | 22 |

(c) 2000-2011 Ingenuity Systems, Inc. All rights reserved.

## Top Bio Functions

### Diseases and Disorders

| Name | p-value | # Molecules |
|---|---|---|
| Cardiovascular Disease | 2.63E-06 - 5.68E-03 | 31 |
| Inflammatory Response | 3.34E-06 - 5.98E-03 | 15 |
| Cancer | 3.67E-06 - 5.25E-03 | 41 |
| Gastrointestinal Disease | 3.67E-06 - 5.07E-03 | 48 |
| Reproductive System Disease | 4.45E-05 - 3.57E-03 | 28 |

### Molecular and Cellular Functions

| Name | p-value | # Molecules |
|---|---|---|
| Cellular Movement | 1.97E-11 - 5.41E-03 | 34 |
| Cell-To-Cell Signaling and Interaction | 6.44E-10 - 6.28E-03 | 33 |
| Cellular Growth and Proliferation | 3.49E-09 - 5.98E-03 | 44 |
| Cellular Development | 4.35E-09 - 5.98E-03 | 39 |
| Cell Death | 4.43E-08 - 6.37E-03 | 37 |

### Physiological System Development and Function

| Name | p-value | # Molecules |
|---|---|---|
| Tissue Development | 6.44E-10 - 5.91E-03 | 35 |
| Tumor Morphology | 2.55E-08 - 3.61E-03 | 16 |
| Connective Tissue Development and Function | 3.16E-06 - 4.74E-03 | 15 |
| Skeletal and Muscular System Development and Function | 7.32E-06 - 5.98E-03 | 19 |
| Hematological System Development and Function | 2.39E-05 - 5.98E-03 | 17 |

(c) 2000-2011 Ingenuity Systems, Inc. All rights reserved.

## Top Canonical Pathways

| Name | p-value | Ratio |
|---|---|---|
| Caveolar-mediated Endocytosis Signaling | 1.24E-03 | 4/85 (0.047) |
| p53 Signaling | 3.53E-03 | 4/96 (0.042) |
| Role of Tissue Factor in Cancer | 5.43E-03 | 4/114 (0.035) |
| NRF2-mediated Oxidative Stress Response | 6.61E-03 | 5/193 (0.026) |
| HER-2 Signaling in Breast Cancer | 1.4E-02 | 3/81 (0.037) |

## Top Molecules

### Fold Change up-regulated

| Molecules | Exp. Value | Exp. Chart |
|---|---|---|
| SERPINE2 | ↑12.790 | |
| HSPA6* | ↑10.220 | |
| IL11 | ↑8.580 | |
| CTGF | ↑8.340 | |
| TGFBI | ↑6.820 | |
| SERPINE1* | ↑6.700 | |
| LARP6 | ↑5.400 | |
| PRSS33 | ↑4.970 | |
| FAS* | ↑4.640 | |
| JAG1* | ↑4.100 | |

### Fold Change down-regulated

(c) 2000-2011 Ingenuity Systems, Inc. All rights reserved.

INGENUITY® SYSTEMS

| Molecules | Exp. Value | Exp. Chart |
|---|---|---|
| PRR15L | ↓6.330 | |
| AGR3 | ↓4.490 | |
| SAMD12 | ↓3.910 | |
| ASCL1 | ↓3.480 | |
| RNF144B | ↓3.210 | |
| FBP1 | ↓3.190 | |
| S100A8 | ↓3.090 | |
| DOK7 | ↓3.010 | |
| SLC26A2 | ↓2.760 | |
| TSGA10 | ↓2.730 | |

(c) 2000-2011 Ingenuity Systems, Inc. All rights reserved.

## Top My Lists

| Name | p-value | Ratio |
|---|---|---|

## Top My Pathways

| Name | p-value | Ratio |
|---|---|---|

## Top Tox Lists

| Name | p-value | Ratio |
|---|---|---|
| Renal Necrosis/Cell Death | 1.03E-03 | 8/314 (0.025) |
| Increases Liver Hepatitis | 1.94E-03 | 2/10 (0.2) |
| Increases Damage of Mitochondria | 1.94E-03 | 2/11 (0.182) |
| p53 Signaling | 3.81E-03 | 4/95 (0.042) |
| Increases Depolarization of Mitochondria and Mitochondrial Membrane | 5.04E-03 | 2/17 (0.118) |

(c) 2000-2011 Ingenuity Systems, Inc. All rights reserved.

INGENUITY® SYSTEMS

## Top Tox Functions

### Assays: Clinical Chemistry and Hematology

| Name | p-value | # Molecules |
| --- | --- | --- |
| Increased Levels of Alkaline Phosphatase | 2.09E-03 - 2.09E-03 | 3 |
| Increased Levels of Potassium | 1.34E-02 - 1.34E-02 | 1 |
| Increased Levels of ALT | 3.31E-02 - 3.31E-02 | 1 |
| Increased Levels of AST | 3.96E-02 - 3.96E-02 | 1 |
| Increased Levels of LDH | 5.89E-02 - 5.89E-02 | 1 |

### Cardiotoxicity

| Name | p-value | # Molecules |
| --- | --- | --- |
| Cardiac Damage | 2.65E-04 - 1.34E-02 | 3 |
| Cardiac Hypertrophy | 4.25E-03 - 1.94E-01 | 6 |
| Cardiac Dysfunction | 5.68E-03 - 5.68E-03 | 2 |
| Cardiac Fibrosis | 6.37E-03 - 2.37E-01 | 2 |
| Heart Failure | 1.03E-02 - 5.97E-02 | 5 |

### Hepatotoxicity

| Name | p-value | # Molecules |
| --- | --- | --- |
| Liver Adhesion | 1.33E-04 - 1.33E-04 | 2 |
| Liver Damage | 1.18E-03 - 8.84E-02 | 6 |
| Liver Hepatitis | 2.78E-03 - 1.83E-01 | 3 |
| Liver Cirrhosis | 3.53E-03 - 3.53E-03 | 4 |
| Liver Cholestasis | 4.43E-03 - 5.25E-02 | 2 |

(c) 2000-2011 Ingenuity Systems, Inc. All rights reserved.

**Nephrotoxicity**

| Name | p-value | # Molecules |
| --- | --- | --- |
| Renal Nephritis | 2.82E-03 - 4.04E-02 | 3 |
| Renal Necrosis/Cell Death | 7.93E-03 - 8.39E-02 | 8 |
| Kidney Failure | 1.34E-02 - 1.20E-01 | 2 |
| Renal Proliferation | 2.18E-02 - 2.18E-02 | 2 |
| Renal Tubule Injury | 1.03E-01 - 1.03E-01 | 2 |

(c) 2000-2011 Ingenuity Systems, Inc. All rights reserved.

# IPA® Summary of Analysis

Analysis Name: slug_day4_fold=2_adjpval=0.01
Analysis Creation Date: 2011-05-23
IPA version: 9.0 (Release Date: 2011-05-20)
Content version: 3210 (Release Date: 2011-05-17)

## Analysis settings

View

Reference set: Human Genome U133 Plus 2.0 Array

Relationship to include: Direct and Indirect

Includes Endogenous Chemicals

Optional Analyses: My Pathways My List

Filter Summary:

Consider only relationships where

(confidence = Experimentally Observed) AND

(data sources = BIND OR BIOGRID OR ClinicalTrials.gov OR Cognia OR DIP OR Gene Ontology (GO) OR GVK Biosciences OR Ingenuity Expert Findings OR Ingenuity ExpertAssist Findings OR INTACT OR Interactome studies OR Kyoto Encyclopedia of Genes and Genomes (KEGG) OR MINT OR MIPS OR Obesity Gene Map Database OR TarBase)

## Top Networks

| ID | Associated Network Functions | Score |
|---|---|---|
| 1 | Cancer, Cellular Development, Tumor Morphology | 37 |
| 2 | Cancer, Cell-To-Cell Signaling and Interaction, Cellular Function and Maintenance | 36 |
| 3 | Cell Cycle, Cancer, Cellular Assembly and Organization | 36 |

(c) 2000-2011 Ingenuity Systems, Inc. All rights reserved.

| | | |
|---|---|---|
| 4 | Gene Expression, Cell-mediated Immune Response, Cellular Development | 36 |
| 5 | Cancer, Reproductive System Disease, Cellular Growth and Proliferation | 34 |

(c) 2000-2011 Ingenuity Systems, Inc. All rights reserved.

## Top Bio Functions

### Diseases and Disorders

| Name | p-value | # Molecules |
|---|---|---|
| Cancer | 2.56E-14 - 1.24E-02 | 367 |
| Gastrointestinal Disease | 7.15E-08 - 1.24E-02 | 370 |
| Genetic Disorder | 7.15E-08 - 1.14E-02 | 583 |
| Reproductive System Disease | 7.76E-07 - 1.14E-02 | 206 |
| Antimicrobial Response | 7.40E-06 - 7.10E-03 | 21 |

### Molecular and Cellular Functions

| Name | p-value | # Molecules |
|---|---|---|
| Cellular Movement | 2.45E-10 - 1.17E-02 | 173 |
| Cell Death | 6.13E-07 - 1.24E-02 | 266 |
| Cell Morphology | 9.70E-07 - 9.99E-03 | 81 |
| Gene Expression | 6.23E-06 - 1.14E-02 | 190 |
| Cellular Development | 1.31E-05 - 1.24E-02 | 196 |

### Physiological System Development and Function

| Name | p-value | # Molecules |
|---|---|---|
| Organ Development | 1.16E-05 - 1.05E-02 | 101 |
| Reproductive System Development and Function | 1.16E-05 - 1.05E-02 | 21 |
| Skeletal and Muscular System Development and Function | 1.71E-04 - 1.24E-02 | 49 |
| Embryonic Development | 3.01E-04 - 1.19E-02 | 61 |
| Connective Tissue Development and Function | 3.27E-04 - 1.17E-02 | 72 |

(c) 2000-2011 Ingenuity Systems, Inc. All rights reserved.

## Top Canonical Pathways

| Name | p-value | Ratio |
|---|---|---|
| Interferon Signaling | 6.67E-07 | 12/36 (0.333) |
| Neuregulin Signaling | 6.37E-04 | 15/102 (0.147) |
| Mechanisms of Viral Exit from Host Cells | 1.32E-03 | 9/45 (0.2) |
| Non-Small Cell Lung Cancer Signaling | 1.44E-03 | 12/79 (0.152) |
| Activation of IRF by Cytosolic Pattern Recognition Receptors | 2.38E-03 | 11/72 (0.153) |

## Top Molecules

### Fold Change up-regulated

| Molecules | Exp. Value | Exp. Chart |
|---|---|---|
| IGFBP7* | ↑13.820 | |
| SRPX | ↑11.730 | |
| CTGF | ↑9.990 | |
| EDIL3 | ↑8.680 | |
| MLLT11 | ↑8.650 | |
| FHL1* | ↑8.630 | |
| CCL26 | ↑7.670 | |
| SERPINE2 | ↑7.510 | |
| MMP13 | ↑6.970 | |
| BCAT1 | ↑6.960 | |

### Fold Change down-regulated

(c) 2000-2011 Ingenuity Systems, Inc. All rights reserved.

| Molecules | Exp. Value | Exp. Chart |
|---|---|---|
| ZNF165 | ↓22.360 | |
| SLC6A14 | ↓16.860 | |
| CEACAM1 (includes others)* | ↓14.740 | |
| SAMD12 | ↓14.190 | |
| LAMP3 | ↓12.550 | |
| SLC27A2* | ↓10.790 | |
| ENPP5* | ↓10.560 | |
| AQP3* | ↓10.180 | |
| GPR160 | ↓10.090 | |
| CLDN3* | ↓9.730 | |

(c) 2000-2011 Ingenuity Systems, Inc. All rights reserved.

## Top My Lists

| Name | p-value | Ratio |
|---|---|---|

## Top My Pathways

| Name | p-value | Ratio |
|---|---|---|

## Top Tox Lists

| Name | p-value | Ratio |
|---|---|---|
| Renal Necrosis/Cell Death | 6.99E-03 | 33/314 (0.105) |
| Increases Depolarization of Mitochondria and Mitochondrial Membrane | 2.09E-02 | 4/17 (0.235) |
| Liver Proliferation | 3.6E-02 | 15/133 (0.113) |
| NRF2-mediated Oxidative Stress Response | 3.88E-02 | 21/237 (0.089) |
| Increases Transmembrane Potential of Mitochondria and Mitochondrial Membrane | 4.49E-02 | 7/50 (0.14) |

(c) 2000-2011 Ingenuity Systems, Inc. All rights reserved.

INGENUITY® SYSTEMS

## Top Tox Functions

### Assays: Clinical Chemistry and Hematology

| Name | p-value | # Molecules |
|---|---|---|
| Increased Levels of Alkaline Phosphatase | 4.39E-02 - 6.89E-02 | 7 |
| Decreased Levels of Albumin | 5.91E-02 - 5.91E-02 | 2 |
| Increased Levels of Albumin | 6.89E-02 - 6.89E-02 | 1 |
| Increased Levels of ALT | 3.00E-01 - 3.00E-01 | 1 |

### Cardiotoxicity

| Name | p-value | # Molecules |
|---|---|---|
| Cardiac Hypoplasia | 1.41E-02 - 1.41E-02 | 3 |
| Cardiac Fibrosis | 6.89E-02 - 1.23E-01 | 3 |
| Cardiac Necrosis/Cell Death | 6.89E-02 - 2.69E-01 | 14 |
| Cardiac Arrythmia | 1.33E-01 - 1.00E00 | 5 |
| Cardiac Damage | 1.33E-01 - 6.05E-01 | 3 |

### Hepatotoxicity

| Name | p-value | # Molecules |
|---|---|---|
| Liver Cirrhosis | 4.75E-03 - 1.95E-01 | 15 |
| Liver Fibrosis | 5.31E-03 - 4.35E-01 | 13 |
| Hepatocellular Carcinoma | 9.51E-03 - 9.51E-03 | 24 |
| Liver Adhesion | 1.36E-02 - 3.49E-01 | 3 |
| Liver Hematopoiesis | 1.36E-02 - 3.94E-01 | 3 |

### Nephrotoxicity

(c) 2000-2011 Ingenuity Systems, Inc. All rights reserved.

| Name | p-value | # Molecules |
|---|---|---|
| Renal Necrosis/Cell Death | 3.58E-03 - 6.05E-01 | 33 |
| Renal Dysplasia | 2.59E-02 - 6.89E-02 | 2 |
| Glomerular Injury | 6.89E-02 - 6.89E-02 | 1 |
| Renal Hypoplasia | 6.89E-02 - 6.89E-02 | 1 |
| Renal Nephritis | 6.89E-02 - 1.00E00 | 7 |

(c) 2000-2011 Ingenuity Systems, Inc. All rights reserved.
