# Supplementary material for: The Transcription Factors Snail and Slug Activate the Transforming Growth Factor-Beta Signaling Pathway in Breast Cancer
Source: PLoS One. 2011 Oct 20;6(10):e26514. doi: 10.1371/journal.pone.0026514 (PMC3197668; doi:10.1371/journal.pone.0026514)
Supplement: Table S1 — Microarray genes changing over time with fold change>2, p-value<0.01 (adjusted), following Snail and Slug expression. Probes that displayed a fold change of two-fold or greater in either direction, along with adjusted p-values less than 0.01 following Snail and Slug expression. (PDF) [file pone.0026514.s007.pdf]

TABLE S1

Day	Snail Vs Control	Slug Vs Control	Common (Snail and Slug)	Snail specific	Slug specific
1	975 (795 down +180 up)	107(46 down+ 61 up)	94	881	13
2	1658 (1281 down+377 up)	134(69 down+ 65 up)	111	1547	23
4	1559 (1210 down+349 up)	1551(1305 down+ 246 up)	886	673	665
