# Supplementary material for: The Transcription Factors Snail and Slug Activate the Transforming Growth Factor-Beta Signaling Pathway in Breast Cancer
Source: PLoS One. 2011 Oct 20;6(10):e26514. doi: 10.1371/journal.pone.0026514 (PMC3197668; doi:10.1371/journal.pone.0026514)
Supplement: Table S2 — List of primers used in study. (PDF) [file pone.0026514.s008.pdf]

Table S2

RT-PCR primers	
CLDN7_RT_F	AGACTTTTATAACCCTTTGATCCCT
CLDN7_RT_R	GTACCCAGCCTTGCTCTCATT
CTGF_RT_F	TAGAGAAGCAGAGCCGCCT
CTGF_RT_R	AGAATTTAGCTCGGTATGTCTTCAT
SPARC_RT_F	TGTGACCTGGACAATGACAAGT
SPARC_RT_R	AAAGAGAGAATCCGGTACTGTG
TAGLN_RT_F	AAGAATGATGGGCACTACCGT
TAGLN_RT_R	AGCCCTCTCCGCTCTAACT
CLDN4-RT-F	GCAGTGCAAGGTGTACGACTC
CLDN4-RT-R	GCGGATTGTAGAAGTCTTGGA

ChIP Primers	
TGFBR2_ChIP_AF	CCCTAGCAAGAAAGGAAATTTGAA
TGFBR2_ChIP_AR	AGTCACCTGAATGCTTGTGCTT
TGFBR2_ChIP_1F	TTCTGATCTACTAGGGAAAACGT
TGFBR2_ChIP_1R	AGCCCCTCCGAGAGCTTT
TGFBR2_ChIP_2F	ATCCCACCGCACGTTCAGAAGT
TGFBR2_ChIP_2R	GACTGTCAAGCGCAGCGGAGA
TGFBR2_ChIP_3F	ACGCCTTGGCATGGATGGAT
TGFBR2_ChIP_3R	AGAACCATCAACTCCCTGAAGA
TGFBR2_ChIP_BF	TGAAGGTGGCATGGAGATGGA
TGFBR2_ChIP_BR	AAAAACGCCAAGAACGAAACACTA
